# Identifying the therapeutic potential of niclosamide in overcoming IFN-gamma dependent cancer immune evasion in the tumor microenvironment

**DOI:** 10.3389/fimmu.2026.1761715

**Published:** 2026-03-16

**Authors:** Yue Zhang, En Cai

**Affiliations:** Department of Biological Sciences, Carnegie Mellon University, Pittsburgh, PA, United States

**Keywords:** cancer immunotherapy, cancer stem cells, IFN-γ, immune evasion, niclosamide, PD-L1, STAT1, STAT3

## Abstract

**Introduction:**

Tumor cells frequently develop immune resistance through interferon-γ (IFN-γ)–induced PD-L1 expression, acquisition of cancer stem cell (CSC)–like features, and adaptation to hypoxia within the tumor microenvironment (TME). Although IFN-γ activates both STAT1 and STAT3, how these pathways interact to regulate immune evasion under hypoxia remains unclear.

**Methods:**

Using the MC38 murine colorectal cancer model and T cell–tumor spheroid co-culture assays, we examined how IFN-γ signaling through STAT1 and STAT3 regulates PD-L1 expression, CSC plasticity, and cytotoxic T cell function under normoxic and hypoxic conditions. Pharmacologic inhibitors and siRNA-mediated knockdown were used to dissect pathway function. Niclosamide, an FDA-approved anthelmintic, was evaluated as a dual STAT1/STAT3 inhibitor.

**Results:**

IFN-γ primarily induced PD-L1 expression through STAT1 activation, whereas CSC plasticity was associated with STAT3 signaling. STAT1 and STAT3 displayed reciprocal regulation, whereby inhibition of one enhanced activation of the other. Niclosamide effectively inhibited phosphorylation of both STAT1 and STAT3, resulting in suppressed PD-L1 upregulation, reduced CSC enrichment, and partial inhibition of hypoxia-induced HIF-1α expression. In co-culture assays, Niclosamide enhanced T cell infiltration, reduced exhaustion under hypoxic conditions, and improved T cell–mediated tumor killing.

**Discussion:**

These findings identify Niclosamide as a potent dual STAT1/STAT3 inhibitor capable of reversing IFN-γ– and hypoxia-driven immune evasion. Repurposing Niclosamide may represent a promising strategy to enhance the efficacy of immune checkpoint blockade in solid tumors.

## Introduction

Over the past decade, cancer immunotherapy, which aims to restore immune function within the tumor microenvironment (TME), has achieved remarkable success in cancer treatment (1, 2). Therapies like immune checkpoint blockade (ICB) have demonstrated durable responses in some patients with advanced malignancies. Nevertheless, only a subset of patients (around 30%) with certain solid tumors benefits from ICBs, while most patients exhibit minimal response to these treatments (3–5). Understanding the mechanisms of immune evasion and identifying new therapeutic targets remain critical for improving treatment outcomes.

Tumor cells employ multiple strategies to escape immune surveillance. One major mechanism is programmed cell death ligand 1 (PD-L1) upregulation, which inhibits cytotoxic T cell (CTL) function (6, 7). Another mechanism is the development of cancer stem cell (CSC)–like properties, which confer resistance to therapy and drive recurrence (8, 9). In addition, hypoxia is a hallmark of the TME that profoundly reshapes cellular metabolism, promotes PD-L1 expression via hypoxia-inducible factors (HIFs), and suppresses CTL and NK cell functions (10).

During immune responses, T cells release type II interferon (IFN-γ), which is a glycosylated protein that facilitates tumor rejection by modulating systemic anti-tumor immunity (11, 12). However, studies reveal a dichotomous nature of IFN-γ: while it enhances immune functions, it also helps cancer cells evade immune attacks. Specifically, IFN-γ stimulation has been shown to increase cytoplasmic expression of PD-L1 (13, 14) and elevate cancer cell stemness (15, 16). Both the PD-1/PD-L1 axis and CSCs play crucial roles in enabling tumor cells to escape anti-tumor immunity across various cancers (17–19). IFN-γ activates downstream Janus kinase/signal transducer and activator of transcription (JAK/STAT) signaling, primarily STAT1 and STAT3, which regulate overlapping gene networks involved in immune evasion (20–22). Therefore, there is an urgent need for therapeutic strategies that inhibit IFN-γ’s effects on cancer immune evasion while preserving its role in facilitating CTL and natural killer (NK) cells in eliminating tumors.

Here, we examined the fine-tuning of the IFN/JAK/STAT pathway under normoxic and hypoxic condition, and identified the specific STAT pathway that drive PD-L1 upregulation and CSC transformation, respectively. Using a mouse colon cancer model, our study reveals a reciprocal relationship between STAT1 and STAT3 activities in cancer cells (MC38). Specifically, inhibition of STAT1 signaling leads to a significant enhancement in STAT3 activation, and conversely, blocking STAT3 activity results in increased STAT1 signaling. Both transcription factors, STAT1 and STAT3, regulate overlapping sets of target genes, many of which play crucial roles in immune evasion mechanisms. This dynamic interplay suggests that the balance between STAT1 and STAT3 could be a critical factor in shaping the immune microenvironment within tumors, potentially influencing cancer progression and resistance to immune-mediated therapies. Furthermore, in evaluating pharmacological methods to block STAT1/STAT3 activities, we identified an anthelmintic drug, Niclosamide, which inhibits the activity of both STAT1 and STAT3, and partially blocks the upregulation of hypoxia-inducible factor 1α (HIF-1α) protein induced by hypoxia. The co-culture study of primary murine T cells and 3D tumor spheroids model further confirmed that Niclosamide could enhance T cell infiltration and reduce T cell exhaustion under a hypoxic TME. Together, our study reveals Niclosamide as a promising candidate for repurposing in cancer immunotherapy to overcome IFN-γ and hypoxia-induced immune evasion.

## Methods

### Cell culture and compounds

MC38 and MC38-OVA cell lines were gifts from Robert Eil lab at OHSU. Both MC38 and MC38-OVA cell line were maintained in DMEM Medium (Gibco, Gaithersburg, MD, USA), supplemented with 10% fetal bovine serum (FBS) and 1% penicillin/streptomycin. Primary OT1 mouse T cells were prepared from lymph nodes and spleens of OT-I TCR transgenic mice (6–10 weeks old), and were maintained in RPMI-1640 Medium (Gibco, Gaithersburg, MD, USA), supplemented with 10% fetal bovine serum (FBS) and 1% penicillin/streptomycin and 50 *μ*M *β*-mercaptoethanol (complete RPMI). Splenocytes were incubated in complete RPMI with 100 ng/mL SIINFEKL peptide for 30 minutes at 37 °C and then washed three times. Lymphocytes and splenocytes were then mixed at 1:1 ratio at 2 million cells/mL in complete RPMI and maintained at 37 °C 2% CO_2_. Interleukin-2 (HIL2-RO, Roche) were added to the cell culture on day 2 at a final concentration of 10 U/mL. Cells were replenished with fresh media and IL-2 every 2 days and used from day 4 to day 7. Mouse IFN gamma Recombinant Protein were purchased from MilliporeSigma (Sigma-Aldrich, USA). STAT1 inhibitor Fludarabine (F-ara-A, NSC 118218) (MedChemExpress, Cat. No.: HY-B0069) and STAT3 inhibitor Niclosamide (BAY2353, MedChemExpress, Cat. No.: HY-B0497) were purchased from MedChemExpress (Monmouth Junction, NJ, USA).

For cell cultured under hypoxic conditions, cells were maintained in the Heracell™ VIOS 160i Tri-Gas CO2 Incubator from Thermo Fisher Scientific (Waltham, MA), and nitrogen gas were connected to the incubator to make the oxygen concentration down to 1.5% inside the chamber. For normoxia condition, the oxygen concentration is 20%, as detected by oxygen sensor.

### Animals

C57BL/6-Tg(TcraTcrb)1100Mjb/J (OT-1) mice (6–8 weeks of age, both sexes) were purchased from the Jackson Laboratory. Mice were housed under specific pathogen–free conditions with controlled temperature, humidity, and a 12-h light/dark cycle, with food and water provided ad libitum.

### Euthanasia and tissue collection

Mice were euthanized using CO_2_ inhalation (gradual-fill method) followed by cervical dislocation to ensure death, in accordance with AVMA guidelines. After euthanasia, peripheral lymph nodes were aseptically harvested for downstream T-cell isolation.

### All animal-related studies have adhered to the ARRIVE guidelines

#### Primary CD8^+^ T-cell Isolation

Single-cell suspensions were prepared from harvested lymph nodes by mechanical dissociation through a 70-µm cell strainer. Red blood cells were removed using ACK lysing buffer when necessary. CD8^+^ T cells were isolated using a negative-selection magnetic bead kit (stem cell), according to the manufacturer’s protocol. Purified primary CD8^+^ T cells were used immediately for co-culture experiments with tumor spheres.

#### Formation of co-culture tumor spheroids

MC38OVA (or MC38) tumor spheroids were generated by seeding 1x104 cells per well on Costar ultra-low attachment (Corning) round bottom 96 wells plates in the 3D Tumorsphere Medium XF (PromoCell). 7 days later, 1x105 total or CD8 sorted mouse primary T cells were labeled with ViaFluor^®^ 650 SE Cell Proliferation Dye, then added with the spheroids to perform co-culture. Spheroids were gently resuspended and left to sediment to the bottom of the Eppendorf tube. These steps were repeated 2 times with PBS in order to wash the spheroids from the non-infiltrating immune cells. Spheroids were then break down mechanically to obtain a single cell suspension for further analyzed by flow cytometry.

#### IFN-γ, fludarabine and niclosamide treatment

**Figure 1:**
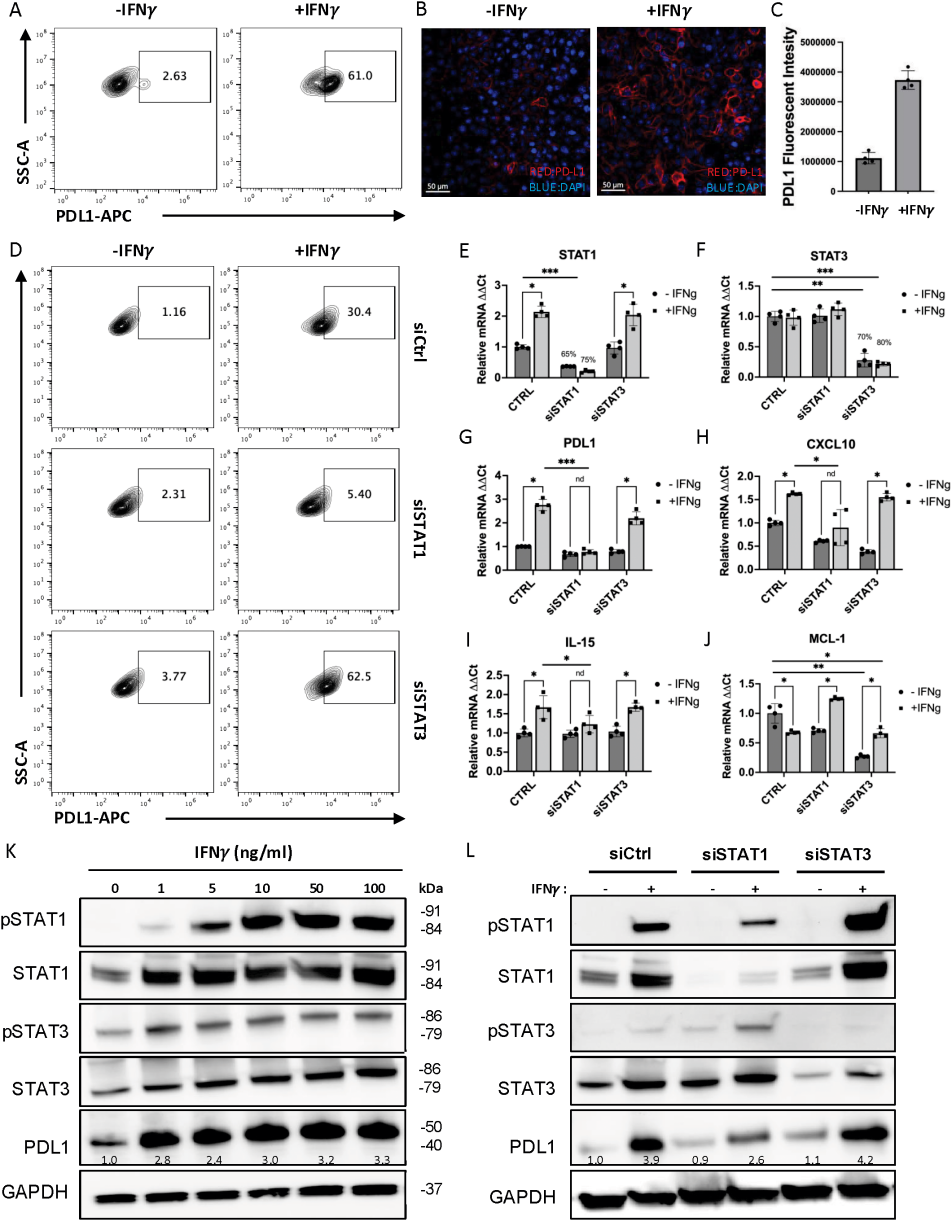
IFNγ up-regulate PD-L1 expression in MC38 cells through IFN-STAT1 signaling. PD-L1 expression on MC38 cell surface was significantly increased in the presence of IFN-γ analyzed by flow cytometry **(A)** and by immune fluorescent staining **(B-C)** at 24 hours. **(D)** When treated with siRNA of STAT1, the up-regulation of PD-L1 was significantly reduced, while siRNA of STAT3 has an opposite effect. **(E, F)** The small interfering RNA reaches a sufficient knock down of STAT1 and STAT3, as shown by real-time PCR assay. **(G-I)** The mRNA of STAT1 target genes such as CXCL10 and IL-15 shown similar trend of regulation by IFN-γ as PD-L1. **(J)** The mRNA of STAT3 target gene, MCL-1, shown totally different regulation under normal condition and under treatment of siRNAs. **(K, L)** The expression of STAT1/STAT3 normal and phosphorylated proteins, and PD-L1 proteins in MC38 cell lines were measured by Western blotting with different dose of IFN-γ stimulation **(K)** or after knocking down of STAT1 (siSTAT1) or STAT3 (siSTAT3) **(L**). The number on the image indicates the relative abundance of PD-L1 protein (fold of control). The results are expressed as the mean ± SEM of triplicate measurements in each group. *p<0.05, **p<0.01, ***p<0.001.

**Figure 2:**
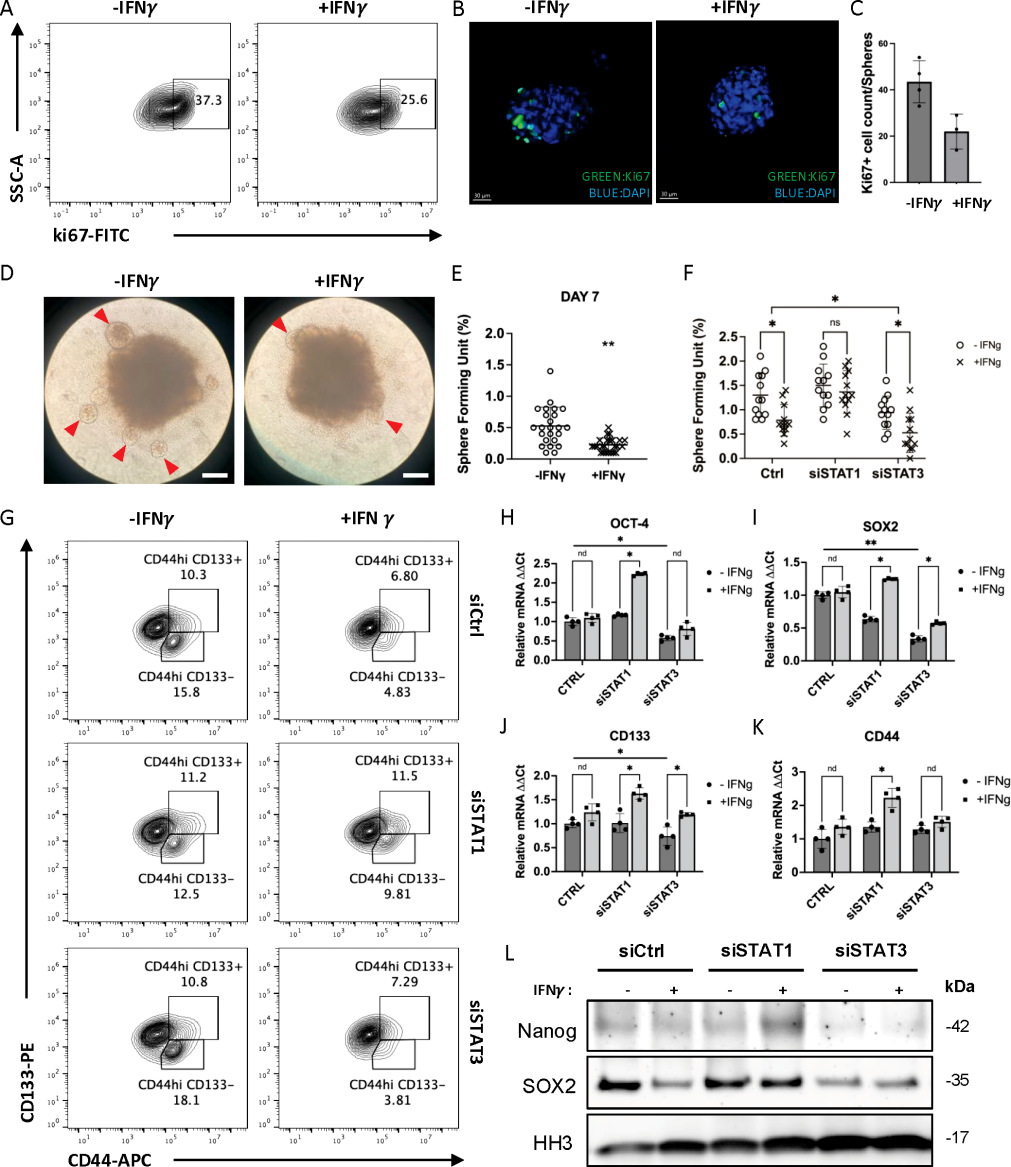
IFN-γ modulate cancer cell stemness in MC38 cells indirectly through IFN-STAT3 pathway. **(A-C)** Ki-67 expression in the MC38 tumor spheres was significantly reduced in the presence of IFN-γ analyzed by flow cytometry and immune fluorescence. **(D, E)** The tumor sphere forming unit induced in the MC38 cells were also reduced in the presence of IFN-γ (Arrowhead: tumor spheroid. Scale bar: 200μm). **(F)** When MC38 cells were pre-treated with siRNA of STAT1/STAT3, the SFU show different changes. **(G)** When MC38 cells were pre-treated with siRNA of STAT1/STAT3, both the CD44^hi^CD133- and CD44^hi^CD133+ cell population show different trend of regulation by IFN-γ. **(H-K)** The mRNA of cancer stem cell markers was measured by real-time PCR. **(L)** The expression of cancer stem cell marker proteins in cell nucleus was measured by Western blot. The results are expressed as the mean ± SEM of triplicate measurements in each group. *p<0.05, **p<0.01, nd, no significant difference.

**Figure 3:**
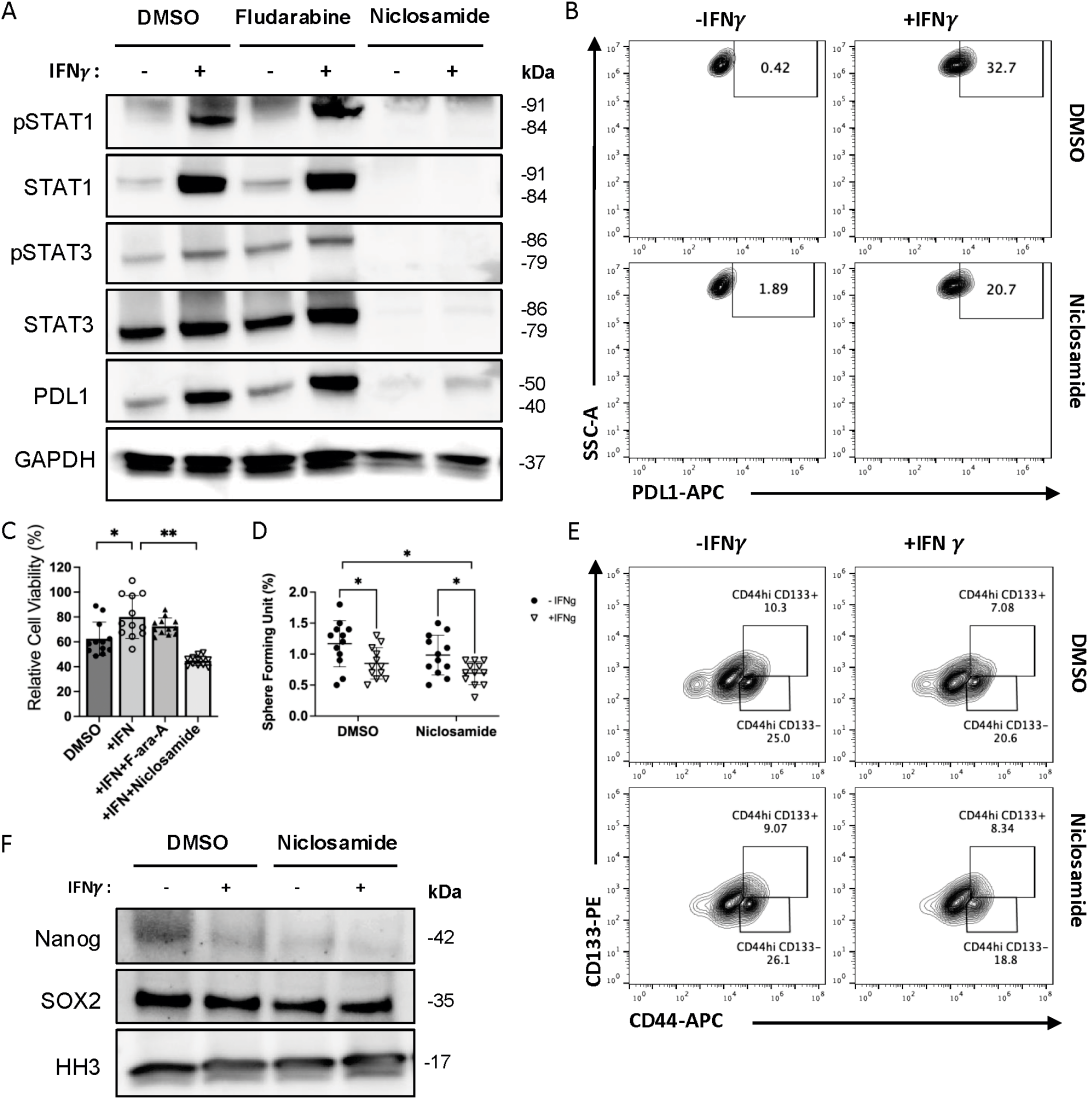
Niclosamide has inhibition effect on both STAT1 and STAT3, which blocks IFN-γ-induced PD-L1 up-regulation in MC38 cells while also reduces CSCs formation. **(A)** The expression of STAT1/STAT3 signaling pathway and PD-L1 proteins in MC38 cell lines with Fludarabine and Niclosamide treatment was measured by Western blot. **(B)** The surface PDL1 expression level was measured by FACS with IFN-γ and Niclosamide treatment. **(C)** The cell viability of MC38 when co-cultured with T cells and pre-treated with Fludarabine (F-ara-A) or Niclosamide were measured by CCK8 assay. **(D)** The sphere forming units induced in MC38 cells was measured with or without Niclosamide treatment. **(E)** The cell population of CD44^hi^CD133+ in MC38 treated with Niclosamide was measured by FACS. **(F)** The expression of cancer stem cell marker proteins in cell nucleus was measured by Western blot. The results are expressed as the mean ± SEM of triplicate measurements in each group. *p<0.05, **p<0.01, ***p<0.001.

**Figure 4:**
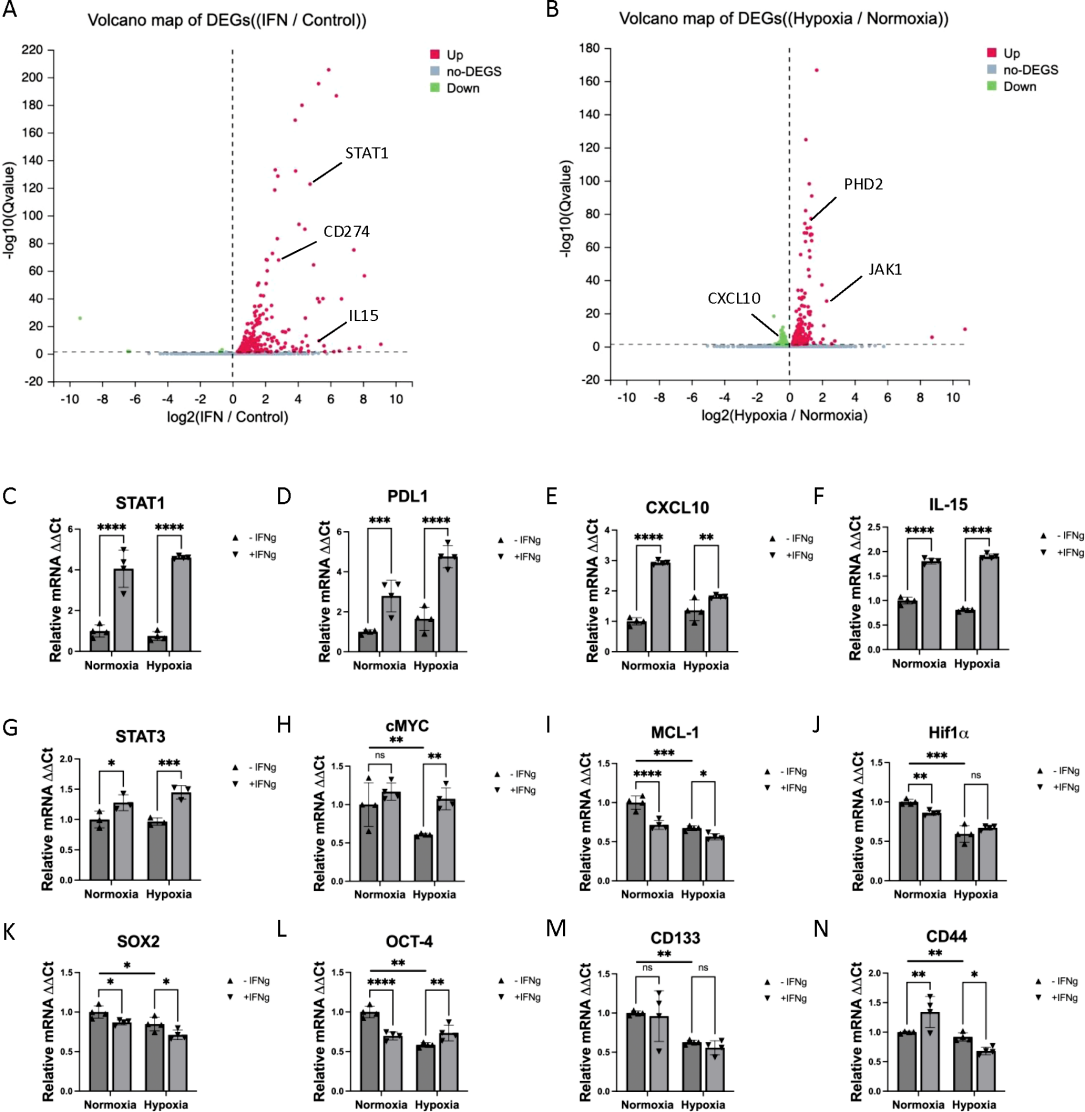
RNA-sequencing analysis from total RNA sample of MC38 cells treated with IFN-γ. **(A)** The volcano map of DEGs from RNA-sequencing analysis comparing IFN-γ-treated group to control group. **(B)** The volcano map of DEGs from RNA-sequencing analysis comparing hypoxia group to normoxia group. **(C-N)** The real-time PCR validation of various genes show changes in the RNA-sequencing. The results are expressed as the mean ± SEM of triplicate measurements in each group. *p<0.05, **p<0.01, ***p<0.001, ****p<0.0001.

5ng/ml IFN-γ treatment for 24 hours were used for most of our experiments, except the experiments with IFN-γ dose and time comparison (**Figures 1K**; **Supplementary Figure 1**). 5 µM of Fludarabine and 0.5 µM niclosamide treatment were used for the experiments shown in **Figures 2**–**4**, these doses were selected based on prior studies demonstrating effective STAT inhibition without overt cytotoxicity.

#### Quantitative real-time PCR

Total RNAs were isolated from cell lysate or mouse tissue with TRIzol reagent (Invitrogen Life Technologies, Carlsbad, CA, USA). The expression levels of multiple mRNAs were detected by quantitative real-time PCR using the High-Capacity cDNA Reverse Transcription Kit (Thermo Fisher Scientific, Waltham, MA, US) and the LightCycler^®^ 480 System (Roche, Indianapolis, IN, USA). Primers used for Quantitative RT-PCR were shown in **Supplementary Table 1**.

#### Western blot

Total proteins were extracted from cultured cells with radio-immunoprecipitation assay (RIPA) buffer (Thermo Fisher Scientific, Waltham, MA, USA) plus fresh protease and phosphatase inhibitors (Roche, Indianapolis, IN, USA). Proteins (10-20μg) were loaded to each lane and separated by sodium dodecyl sulfate polyacrylamide gel electrophoresis. Proteins were then transferred to a 0.45 µm pore size PDVF membrane (Bio-Rad, Hercules, CA, US), followed by immunodetection of target proteins using specific antibodies with chemiluminescent detection. The developed films were quantified with FIJI (ImageJ) software, relative protein abundance was normalized to GAPDH, beta Actin or Histone H3, and then normalized to the first lane (control group), calculated as fold of control. The antibodies used in Western blot were listed in **Supplementary Table 2**.

#### Flow study

Cell suspensions of MC38 or primary T cells were incubated with the different combinations of antibodies (for MC38: PD-L1, CD133, CD44, B7H4, For T cells: CD45, CD8, PD1, Tim3) on ice for 30 minutes. Then the samples were washed and fixed after cell surface staining according to the manufacturer’s description in fixative buffer(eBioscience). All antibodies were purchased from BD Biosciences and flow data were collected on a BD Accuri C6 Plus Flow Cytometer (BD Biosciences). The antibodies used in flow study were also listed in **Supplementary Table 2**.

The data were analyzed using the FlowJo software (Tree Star Inc.).

#### Immunofluorescence

For immunofluorescence, cultured cells or tumor spheres were rinsed with 0.1% TX-100 in PBS (PBST) three times, blocked with 1% goat serum and 2% BSA in PBS at room temperature for 1 h and then incubated with the primary antibody conjugated with fluorescence. Then the slides were washed 3 times with PBST, and mounted with Fluoromount-G™ Mounting Medium (Invitrogen™). Fluorescent images were captured with All-in-One Fluorescence Microscope BZ-X800 (Keyence, Ōsaka, Japan). The primary antibodies used in the immunofluorescence were shown in **Supplementary Table 2**.

### RNA sequencing

RNA-seq of IFN-γ treated MC38 cells was performed by Innomics Inc. (One Broadway, 14th FL, MA) using DNBSEQ Eukaryotic Strand-specific Transcriptome Resequencing. The RNA-seq experiment was performed with a single biological sample per condition: control vs IFN-γ treatment, normoxia vs hypoxia. Alignment of raw sequencing reads was performed using Hisat2 software. The DNBSEQ package was used to find differentially expressed genes, and differentially expressed genes with P < 0.05 were used to perform cluster analysis and enrichment analysis with ClusterProfiler package in the R program.

#### Statistics

Data were presented as mean ± SEM. Statistical analysis was performed with GraphPad Prism 10.0 software (GraphPad Software, San Diego, CA). Comparison between experimental variables was performed using one-way or two-way ANOVA followed by a Tukey *post hoc* test. Significance levels were denoted as *p <0.05, **p < 0.01, or ***p <0.001.

#### Study period

2023. June -2025.September.

## Results

### IFN-γ induced PD-L1 expression through STAT1 pathway

In this study, we used the mouse colon cancer cell line MC38 as a model to study the effects of IFN-γ treatment. Consistent with previous literature reports, we found a significant increase in PD-L1 expression on MC38 cells following treatment with IFN-γ, as shown by both flow cytometry (**Figure 1A**) and immunofluorescent staining images (**Figure 1B, C**). The upregulation of cell surface PD-L1 occurred in a dose and time-dependent manner (**Supplementary Figure 1A, B**). In addition, PD-L1 upregulation was also observed in induced MC38 tumor spheres, which exhibited more CSC features (increased population with CD44+CD133+ expression) compared to adherent MC38 cells (**Supplementary Figure 2A, B**).

To access how IFN-γ treatment affect tumor cell viability, we used a T cell–tumor cell co-culture system. We found treatment with 5 ng/mL IFN-γ enhanced the survival of MC38 cells when co-cultured with primary mouse T cells at a 1:1 ratio. This effect was even more pronounced in tumor spheroids, which exhibited increased resistance to T cell-mediated killing (**Supplementary Figure 2D**). Furthermore, MC38 cells pre-treated with 5 ng/mL IFN-γ showed markedly increased resistance to T cell cytotoxicity during short-term co-culture (30 min to 4 h) (**Supplementary Figure 3A**). Consistently, colon cancer cells exposed to low concentrations of IFN-γ (0.5–5 ng/mL) developed enhanced resistance to T cell killing (at a 1:3 T cell-to-tumor ratio), whereas higher IFN-γ concentrations (10–100 ng/mL) led to decreased viability (**Supplementary Figure 3B**). Therefore, we determined to use IFN-γ concentration at 5 ng/mL for our experiments.

Next, we sought to determine the specific STAT signaling pathway that is responsible for IFN-γ induced PD-L1 upregulation. IFN-γ has been reported to signal through STAT1 and STAT3 (23, 24). However, the primary signal transducer responsible for IFN-γ dependent PD-L1 upregulation varies between different tumor cell lines (25). To evaluate the potential roles of STAT1 and STAT3 activation in mediating PD-L1 protein expression in MC38, we used small interfering RNA (siRNA) to knock down STAT1 and STAT3 and examined their distinct contributions to IFN-γ-induced PD-L1 expression. Flow cytometry results showed that IFN-γ-induced PD-L1 expression was diminished when STAT1 was knocked down, whereas STAT3 knockdown have an opposite effect (**Figure 1D**). A similar effect was observed in MC38 cells that were pre-transfected with siRNAs and then induced into tumor spheres (**Supplementary Figure 2C**). These results suggest that in MC38 cells, IFN-γ upregulate PD-L1 expression through the IFN-STAT1 signaling pathway.

To verify siRNAs knockdown and assess target gene expression levels, we performed real-time PCR. The results confirmed an efficient knockdown of STAT1 (**Figure 1E**) and STAT3 (**Figure 1F**) by siRNA. Classical STAT1 transcriptional targets including CXCL10 and IL-15 was upregulated in response to IFN-γ treatment, and the up-regulation was diminished with the siRNA of STAT1, but not siSTAT3 (**Figures 1G–I**). In comparison, myeloid cell leukemia-1 (MCL-1), a transcriptional target of STAT3, was down-regulated by IFN-γ treatment. Knocking down STAT1 increased MCL-1 in the IFN-γ treated sample (**Figure 1J**). The mRNA levels of these target genes further confirmed efficient knocking down of STAT1 and STAT3. Notably, when examining PD-L1 levels, we found that IFN-γ-induced PD-L1 upregulation is diminished by knocking down STAT1 but not STAT3. To further assess the total protein expression, we performed western blot analysis, which showed that IFN-γ upregulate PD-L1 in a dose-related manner (**Figure 1K**) and that knocking-down of STAT1 with siRNA (siSTAT1) partially blocks the upregulation of PD-L1 induced by IFN-γ (**Figure 1L**). The quantification of the relative band density of these western blot images was present in **Supplementary Figures 1C–L**. Together, these results confirm that IFN-γ treatment of MC38 cells up-regulate PD-L1 expression through the IFN-STAT1 signaling pathway.

Next, we sought to assess the activation levels of STAT1 and STAT3. We performed western blot assays of the phosphorylated STAT1 (pSTAT1), and phosphorylated STAT3 (pSTAT3) and found that both were enhanced by IFN-γ, and the induced pSTAT1 is much stronger compared to pSTAT3 at higher IFN-γ concentrations. This suggest that IFN-γ activate STAT1 much more strongly than STAT3. Interestingly, as we compared western blot results of siSTAT1/3 treated samples with scramble siRNA control (siCtrl), we observed that knocking down STAT1 increased pSTAT3 protein level induced by IFN-γ while knocking down STAT3 increased pSTAT1 level induced by IFN-γ (**Figure 1L**) (**Supplementary Figures 1H–K**). This suggests competitive interactions between pSTAT1 and pSTAT3, likely due to the two transcription factors may compete for shared kinases and promoter binding sites (25). This competition explains that knockdown of STAT3 enhances the increase in PD-L1 expression induced by IFN-γ in western blot (**Figure 1L**) (**Supplementary Figure 1L**). and flow cytometry (**Figure 1D**). Therefore, IFN-γ-dependent PD-L1 upregulation in MC38 can be suppressed by inhibiting the STAT1 signaling pathway, but this also leads to potential side effects of increased STAT3 activity.

#### IFN-γ regulate cancer cell stemness indirectly through pSTAT1/pSTAT3 competitive activity

Parallel to our study on IFN-γ-dependent PD-L1 expression in MC38 cells, we sought to investigate the impact of IFN-γ on mitosis and cell cycle. A recent study using mouse models of breast cancer showed that IFN-γ produced by activated T cells can directly convert non-CSCs to CSCs (16). However, it remains unclear whether this effect is specific to breast cancer models or could be observed across multiple cancer types. Based on evidence that type I interferons (IFNs-I) promote an adaptive yet reversible transcriptional rewiring of cancer cells towards stemness and immune escape (15), we seek to determine how IFN-γ treatment shape the stemness in MC38 cancer cells.

We generated colon cancer spheroids from MC38 cells. We first used Ki-67 to label the active proliferating cells in mix cell population. To our surprise, we found that IFN-γ treatment reduced the number of Ki-67 positive cells in a MC38 tumor spheroids model (**Figures 2A–C**). To access cancer stemness, we measured the number of sphere-forming unit (SFU) in MC38 cells and found it significantly repressed by IFN-γ pre-treatment (**Figure 2D, E**), which suggests a reduction in cancer stemness. Furthermore, the reduction of SFU can be neutralized by STAT1 siRNA but not by STAT3 siRNA (**Figure 2F**). We next assessed cancer cell stemness with cancer stem cell surface markers, including CD44 and CD133, and observed a decrease of both CD44^hi^CD133^+^ and CD44^hi^CD133^-^ populations in MC38 cells treated with IFN-γ (**Figure 2G**). Given that these populations were previously reported to represent colorectal cancer stem cells (26–29), our results suggest that CSC stemness decreases upon IFN-γ treatment. Notably, this reduction was abolished only by knocking down STAT1, but not STAT3 (**Figure 2G**). Accordingly, real-time PCR results demonstrated that IFN-γ can induce CSC markers, including CD44, CD133, OCT4, and SOX2, only when tumor cells were transfected with siSTAT1 (**Figures 2H–K**). Furthermore, western blot results showed that the protein levels of CSC markers, including SOX2 and Nanog, were reduced upon IFN-γ treatment under normal condition, but remain the same or at higher levels with STAT1 knocking down (**Figure 2L**) (**Supplementary Figure 2E, F**). Together, these results showed STAT1 knockdown results in upregulation of CSC-related genes, suggesting that STAT1 activation playing suppression roles for regulating these CSC-related genes. On the other hand, when MC38 cells were transfected with siSTAT3, both the mRNA and protein of the CSC markers were significantly reduced (except CD44 mRNA, since CD44 is a gene involved in cell–cell interactions, cell adhesion and migration, and may involve other regulation mechanisms) (**Figures 2H–L**), suggesting that these CSC markers are strongly regulated by STAT3 (30, 31). The observation that STAT1 knockdown results in upregulation of CSC-related genes, which are known to be strongly regulated by STAT3, provides further evidence supporting the competitive antagonism between pSTAT1 and pSTAT3 as signal transducers and transcriptional factors. Therefore, we conclude that the IFN-γ-induced decrease in CSCs is primarily due to the dominant activation of JAK/STAT1 pathway, which results in increased pSTAT1 that competes with pSTAT3, thereby indirectly suppressing STAT3 signaling. Notably, when STAT1 activity is inhibited, IFN-γ can facilitate the transformation to CSCs through compensatory activation of STAT3 signaling. Therefore, suppressing cancer stemness in the MC38 tumor model requires blocking both STAT1 and STAT3 signaling pathways.

#### Niclosamide inhibits both STAT1 and STAT3 activity

We have demonstrated that inhibiting individual STAT1 or STAT3 signaling pathway activated by IFN-γ cannot prevent immune evasion in MC38. To prevent the over-expression of PD-L1 on tumor cells while also suppress the development of CSCs, inhibition of both STAT1 and STAT3 pathways are required.

Next, we sought pharmacological approaches to inhibit IFN-γ induced STAT signaling. We evaluated small molecule STAT1 inhibitor Fludarabine (F-ara-A, NSC 118218) (MedChemExpress, Cat. No.: HY-B0069) and STAT3 inhibitor Niclosamide (BAY2353, MedChemExpress, Cat. No.: HY-B0497). Fludarabine works by inhibiting the cytokine-induced activation of STAT1 and STAT1-dependent gene transcription in normal resting or activated lymphocytes (32, 33). Niclosamide is reported as a STAT3 inhibitor with an IC50 of 0.25 μM in HeLa cells (34). Both reagents were evaluated in our experiments for blocking STAT1 and STAT3 transcriptional activity respectively.

The western blot result showed that 5μM Fludarabine did not have a sufficient inhibition effect to STAT1 phosphorylation in MC38 cells (**Figure 3A**). Fludarabine exhibits context-dependent STAT1 inhibitory activity, and in our system its overall effectiveness appears limited, potentially due to selective STAT1 inhibition accompanied by compensatory STAT3 activation. In comparison, 0.5μM Niclosamide demonstrated disruption of STAT1 protein expression and phosphorylation while also inhibiting STAT3 protein expression and activity (**Figure 3A**) (**Supplementary Figures 3C–G**). In flow-cytometer assays, Niclosamide partially blocked the up-regulation of cell surface PD-L1 induced by IFN-γ (**Figure 3B**), despite Niclosamide being reported primarily as a STAT3 inhibitor, this effect is opposite compared to STAT3 siRNA (**Figure 1D**). When low dose of IFN-γ (5ng/ml) were used to increase tumor cell viability in a T cell killing assay, Niclosamide could neutralize this effect while Fludarabine could not (**Figure 3C**). Meanwhile, Niclosamide did not affect the effectiveness of IFN-γ in reducing SFU (**Figure 3D**) (**Supplementary Figure 4A, B**), nor in reducing the population of CD44^hi^CD133^+^/CD44^hi^CD133^-^ cell population in MC38 induced tumor spheroids (**Figure 3E**). Additionally, niclosamide reduced PD-L1 expression on the cell surface of these tumor spheroids (<xr rid="sf4">Supplemental **Figure 6C**). In western blot assay, Niclosamide did not affect the downregulation of Nanog, SOX2 proteins induced by IFN-γ (**Figure 3F**) (**Supplementary Figure 3H, I**). These data suggested Niclosamide could disrupt both STAT1 and STAT3 phosphorylation in IFN-γ signaling, and could become a potential method to prevent cancer cell’s immune evasion in combination use with immune-checkpoint blockers.

**Figure 5:**
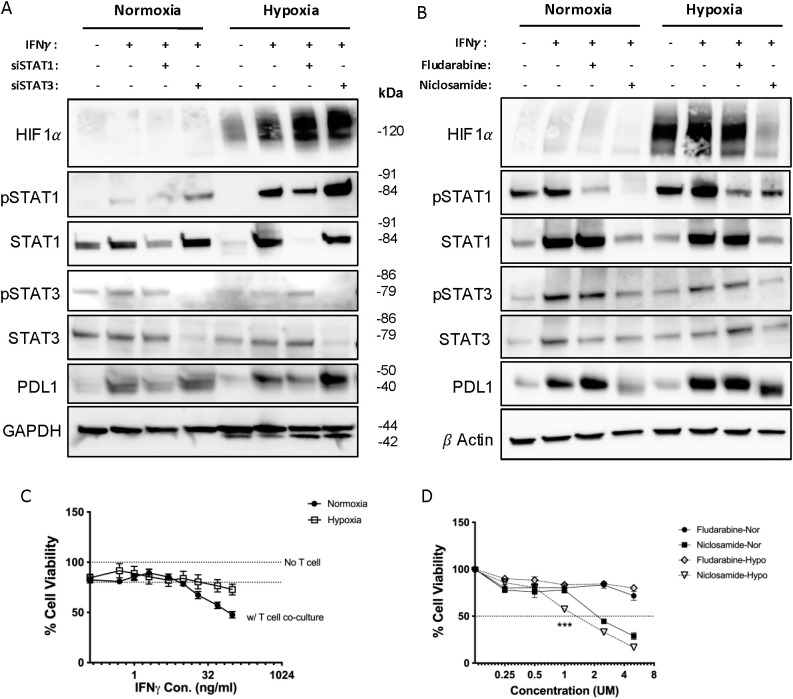
Hypoxia enhances PD-L1 upregulation by IFN-γ, while Niclosamide down-regulates Hif1α under hypoxic conditions. **(A, B)** The expression level of STAT1/STAT3 signaling pathway and PD-L1 proteins in MC38 cell lines with siRNA **(A)** or Fludarabine and Niclosamide treatment **(B)** was measured by Western blot. **(C)** Cell viability of MC38 cells pre-treated with different dose of IFN-γ and co-cultured with primary T cells were measured by CCK8 assay, under normoxia or hypoxic conditions. **(D)** The Cell viability of MC38 cells treated with different dose of Fludarabine or Niclosamide were measured by CCK8 assay, under normoxia or hypoxic conditions. The results are expressed as the mean ± SEM of triplicate measurements in each group. *p<0.05, **p<0.01, ***p<0.001.

**Figure 6:**
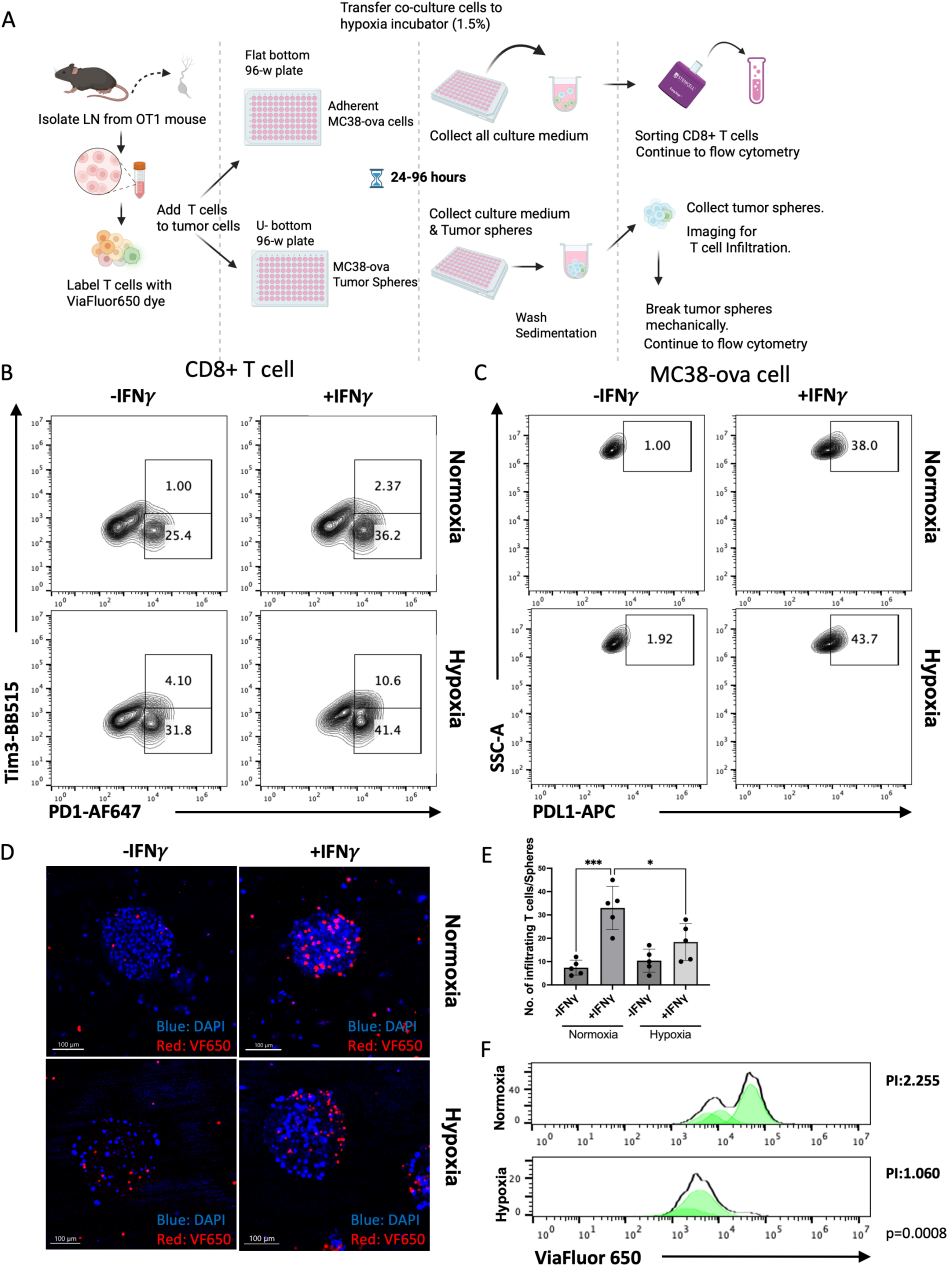
IFN-γ and Hypoxic conditions has synergistic effect on inducing T cell exhaustion, and IFN-γ is essential for T cell infiltration in a tumor-T cells co-culture system. **(A)** Scheme of the protocol used to create co-culture model, using MC38ova adherent cells or tumor-derived spheroids with mouse primary T cells. **(B)** The exhaustion state of CD8+T cells co-cultured with MC38ova cells measured by FACS. **(C)** The expression level of PDL1 on MC38ova cells co-cultured with primary T cells measured by FACS. **(D, E)** The confocal image of tumor-derived spheroids and infiltrating T cells labeled with ViaFluor650 (VF650), with quantification of infiltrating T cell numbers. **(F)** The histogram of ViaFluor650-labeled T cells in the co-culture system measured by flow analysis. Representative ViaFluor 650 dilution histograms showing reduced T cell proliferation under hypoxic conditions. Quantification of proliferation index (PI) across biological replicates is shown on the right; data are presented as mean ± SEM; p-values were calculated using permutation tests. *p<0.05, **p<0.01, ***p<0.001.

#### Delineate the signal network in inflammatory TME under hypoxia

Hypoxic conditions in TME activated a complex signaling network mediated by HIFs, which are closely associated with the JAK/STAT signaling pathways. The hypoxia environment in the solid tumor is well known to mediates resistance to chemotherapy, radiotherapy, and immunotherapy through complex mechanisms (17). To delineate the changes in multiple signaling pathways during IFN-γ treatment under hypoxic conditions, we sequenced total RNA isolated from the following 4 samples: *Ctrl_1_1*: MC38 cells under normal condition, without IFN-γ; *IFN_1_2*: MC38 cells treated with IFN-γ, under normal condition; *Ctrl_2_1*: MC38 cells under hypoxic condition, without IFN-γ; *IFN_2_2*: MC38 cells treated with IFN-γ, under hypoxic condition. The total RNA-sequencing analysis provided insights into the transcriptional landscape of cancer cells that has been modulated by the IFN-γ and hypoxia stimulation (**Figure 4A, B**). Among all the differentially expressed genes (DEGs), we validate a series of genes related to cancer progression and immune response with real-time PCR (**Figures 4C–N**). One of these genes is CD274 (PD-L1), which is significantly up-regulated by IFN-γ alone and further enhanced under hypoxic conditions (**Figure 4D**). STAT1 and its target genes were all up-regulated by IFN-γ in both conditions (**Figures 4C–F**). Notably, the induced CXCL10 (also known as Interferon gamma-induced protein 10, IP-10) level is reduced under hypoxic conditions compared to normoxic conditions (**Figure 4E**), consistent with literature reports that in certain cancer cells, such as colorectal, breast, ovarian, and lung cancer cells, prolonged hypoxia reduces CXCL10 expression, which is linked to the activity of the HIF-1α protein (35). According to quantitative PCR results, STAT3 mRNA was also up-regulated by IFN-γ, but its target genes including MCL-1, and some stem cell marker such as SOX2 and OCT-4 mRNA were down-regulated under hypoxic conditions (**Figures 4G–N**). These results indicate that STAT3 activity may be indirectly modulated under hypoxic conditions and contribute to downstream transcriptional responses. In general, the RNA-seq results further confirms the role of JAK-STAT signaling in regulating potential genes that mediate cancer immune evasion under hypoxic conditions, such as immune checkpoint regulators (CD274) and chemo attractors (CXCL10).

### Hypoxia enhances IFN-γ induced PD-L1 upregulation, which is also blocked by niclosamide

To further investigate the contribution of hypoxia to immune evasion, we performed western blot analysis of various proteins under both conditions. We found that both PD-L1 and pSTAT1 were up-regulated more significantly in hypoxic conditions compared to normoxic conditions (**Figure 5A**) (**Supplementary Figures 5A–F**). More importantly, Niclosamide significantly neutralized the induction of HIF1α under hypoxic conditions, while preserved its ability to block both STAT1 and STAT3 phosphorylation and protein levels (**Figure 5B**) (**Supplementary Figures 5G–L**). The MC38 cells survived better with higher IFN-γ concentration in hypoxic conditions compared to normoxic conditions in a T cell-tumor cell co-culture assay (**Figure 5C**). In a dose-dependent cell viability assay, we found that Niclosamide showed more toxicity to tumor cells under hypoxic conditions (**Figure 5D**). These findings further suggest that Niclosamide could be used as an efficient pharmacological reagent to eliminate IFN-γ-induced immune resistance.

#### IFN-γ induces T cell exhaustion with hypoxia and enhance T cell infiltration in the TME

Given that STATs are also key molecular determinants of T-cell fate and effector function (36), we sought to further study the recruitment and exhaustion progress of T cells in an immunosuppressive tumor microenvironment. We used tumor spheroids induced by MC38-ova cells (transgenic MC38 cell line with stably expressing Ovalbumin) and a T cell-tumor cell co-culture system, to mimic a simplified TME (37) (**Figure 6A**). Using an established protocol of *in vitro* T cell exhaustion (38), we were able to induce mouse primary CD8+ T cells to an early or late exhausted state (**Supplementary Figure 6A**). With the exhausted T markers including PD-1, Tim-3, we found that both early state exhaustion (PD-1+/Tim-3-) and late state exhaustion (PD-1+/Tim-3+) T cells were significantly increased when co-cultured with MC38ova cells that were pre-treated with IFN-γ, and under hypoxic conditions (**Figure 6B**). Meantime, MC38ova cells also showed more induced PD-L1 expression under hypoxic conditions (**Figure 6C**). We found IFN-γ could promote CD8+ T cells infiltration into the tumor spheres significantly, as shown by CD8+ T cells prelabeled with ViaFluor 650 (VF650) (**Figure 6D, E**). We hypothesize this effect is due to CXCL10, a powerful chemokine that acts by binding to its specific receptor, CXCR3, to attract immune cells such as T cells and macrophages to the sites of inflammation. Under hypoxic conditions, the T cell infiltration with IFN-γ stimulation is reduced moderately (**Figure 6D, E**), which is consistent with our finding that CXCL10 expression is also induced to a lesser degree by IFN-γ under hypoxic conditions (**Figure 4E**). In addition, ViaFluor^®^ 650 SE dye (Biotium, Fremont, CA) is a membrane-permeant compound that is converted to fluorescent dye by intracellular esterases and will covalently reacts with amine groups on intracellular protein at the same time, forming fluorescent conjugates that are retained in the cell. In our experiment, T cell proliferation was quantified using a proliferation index calculated from ViaFluor 650 dilution profiles. The CD8+ T cells isolated from tumor spheroid show stronger proliferation index (PI = 2.255) in histogram under normal condition, and weaker proliferation index (PI = 1.060) under hypoxic conditions (**Figure 6F**). Statistical significance between normoxic and hypoxic conditions was assessed using sample-level permutation tests, yielding a p-value of 0.0008.

**Figure 7:**
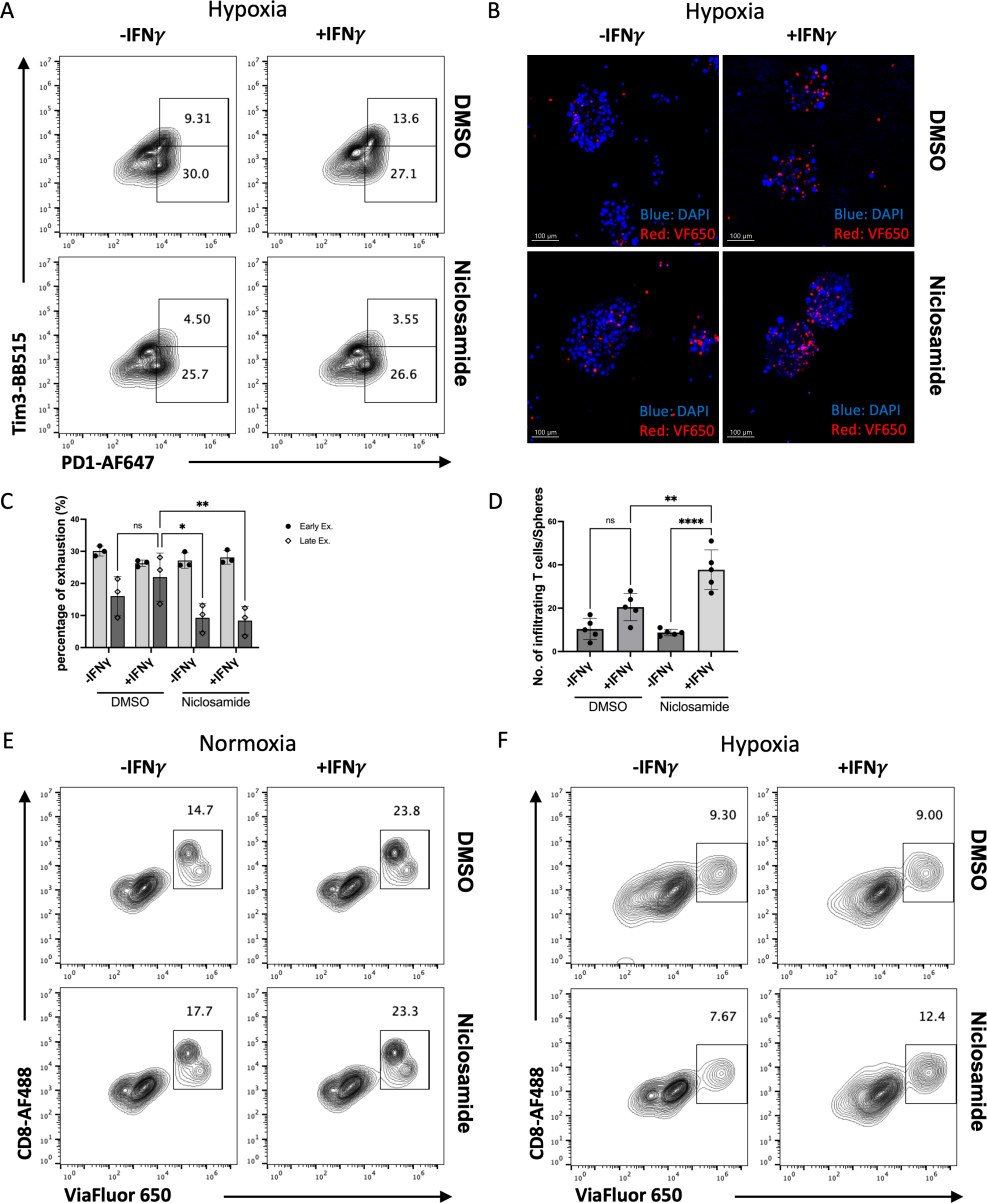
Niclosamide reduces tumor cell immune evasion while preserving IFN-γ’s effect in facilitating T cell infiltration in a tumor spheroids-T cells co-culture system under hypoxic conditions. **(A)** The exhaustion state of CD8+T cells co-cultured with MC38ova cells measured by FACS, pre-treated with or without Niclosamide. **(B)** The confocal image of tumor-derived spheroids and infiltrating T cells labeled with viaFluor650. **(C)** Quantification of the exhausted CD8+ T cells population across biological replicates in panel **(A)** PD1+Tim3- cell population indicate early state exhaustion (Early Ex.) while PD1+Tim3+ indicate late state exhaustion (Late Ex.). **(D)** Quantification of Tumor infiltrating CD8+ T cells in panel **(B, E, F)** The flow analysis of infiltrating T cells labeled with ViaFluor650 under normal or hypoxic conditions, treated with or without Niclosamide. The results are expressed as the mean ± SEM of triplicate measurements in each group. *p<0.05, **p<0.01, ***p<0.001.

In another co-culture assay, when the MC38ova cells were pre-treated with both IFN-γ and Niclosamide, the late state exhausted T cell (PD1+Tim3+) population was less compared to IFN-γ treated only condition (**Figures 7A, C**). In the T cell-tumor spheres co-culture model, although Niclosamide did not change IFN-γ induced CD8+ T cell infiltration under normal condition, but when IFN-γ’s effect was reduced under hypoxic conditions, Niclosamide could partially rescue T cells’ infiltration (**Figures 7B, D–F**). Also, compared to ViaFluor650 labeled CD8+ T cells maintained in media, which divided multiple times in 24hours (PI = 3.31), the CD8+ T cells infiltrated into tumor spheroids show only one time division (PI = 1.29). Niclosamide did not impair the population of total infiltrated CD8+ T cells (CD8+ Infil), nor the sub-population of proliferating T cells that infiltrated (CD8+ proli) (**Supplementary Figures 6B, C**). When we use MC38 tumor spheroids generated from tumor cells that previous knocked down with STAT siRNAs, we found that when co-cultured with cytotoxic T cells, STAT1 knockdown in the tumor spheres impede CD8+ T cell infiltration, while STAT3 knockdown has opposite effect (**Supplementary Figure 7**). Together, the inhibition of both STAT1/STAT3 pathway with Niclosamide not only reduces PD-L1 overexpression induced by IFN-γ, but also rescues the hypoxia induced T cell’s exhaustion and less infiltration related to T cell function and metabolism changes. These results suggest that combining IFN-γ with Niclosamide may enhance therapeutic efficacy in colorectal cancer by mitigating immune evasion.

## Discussion

Both hypoxia and chronic inflammation are well-recognized hallmarks of solid tumor progression (1, 39). These conditions profoundly remodel the tumor microenvironment (TME), driving metabolic reprogramming, immune suppression, and resistance to therapy (17, 18, 40). Recent reviews further emphasize the central roles of STAT family proteins, including STAT1 and STAT3, in orchestrating metabolic reprogramming and transcriptional programs that integrate cytokine signaling, tumor progression, and immune regulation in the TME (41, 42). Hypoxia restricts immune cell infiltration, promotes the accumulation of regulatory T cells (Tregs) and myeloid-derived suppressor cells (MDSCs), and induces PD-L1 expression in both tumor and stromal compartments (17, 18). These adaptive responses converge on central signaling hubs—including HIF-1α, STATs, NF-κB, VEGFA and PI3K–mTOR—which collectively coordinate tumor survival and immune evasion (18, 43). In parallel, inflammatory cells such as tumor-associated macrophages (TAMs) contribute to aberrant and leaky vasculature, further exacerbating intratumoral hypoxia (17, 36, 40, 44, 45).These observations prompted our interest in the interaction between hypoxia and IFN-γ signaling, as IFN-γ and other cytokines secreted by tumor-infiltrating lymphocytes (TILs) robustly activate the JAK–STAT pathway and may synergize with hypoxic stress to shape immune escape mechanisms (21, 43).

In this study, we identify a critical interplay between IFN-γ signaling and hypoxia in regulating tumor immune evasion. We demonstrate that STAT1 predominantly mediates IFN-γ–induced PD-L1 expression, whereas STAT3 promotes cancer stemness, with reciprocal regulation between these pathways (**Figures 1**, **2**). Consistent with these findings, recent publications highlight the multifaceted roles of STAT3 in cancer, including promotion of proliferation, survival, angiogenesis, immunosuppression, and stemness, making it a key mediator of tumor immune evasion and a promising therapeutic target (30, 31, 46). Although IFN-γ potently induces PD-L1 expression in tumor cells, perturbation of IFN-γ signaling triggers compensatory mechanisms that preserve immune resistance. Notably, PD-L1 expression is regulated by multiple pathways in cancer cells, and several mechanistic studies have mapped diverse upstream regulators of PD-L1 beyond IFN-γ signaling, further supporting the complexity of immune checkpoint control (47). Consistent with this finding, selective inhibition of STAT1 or STAT3 alone resulted in compensatory activation of the alternate pathway, underscoring the limitations of single-axis therapeutic strategies targeting the IFN-γ–STAT axis.

We further show that Niclosamide, an FDA-approved anthelmintic drug, functions as a dual inhibitor of STAT1 and STAT3 in the MC38 tumor model. Unlike selective STAT inhibitors, Niclosamide simultaneously suppressed PD-L1 upregulation, reduced cancer stem cell (CSC) enrichment, and partially attenuated hypoxia-induced HIF-1α signaling (**Figure 3**, **Figure 5**). Functionally, Niclosamide restored T cell cytotoxicity, enhanced T cell infiltration, and reduced T cell exhaustion within a hypoxic TME (**Figure 7**). Given its oral bioavailability, low cost, and long-standing clinical safety profile, Niclosamide represents an attractive candidate for rapid clinical translation as an adjuvant to immune checkpoint blockade (ICB). By concurrently targeting STAT1/STAT3 signaling and hypoxia-driven adaptive programs, Niclosamide may expand the therapeutic benefit of ICB in tumors that are otherwise refractory to immunotherapy. It is also important to note that Niclosamide has been reported to exert anticancer effects through multiple signaling pathways beyond JAK–STAT, including Wnt/β-catenin, mTOR, and NF-κB signaling (34, 48, 49). While the present study primarily focuses on the role of Niclosamide in modulating IFN-γ–dependent STAT1 and STAT3 activation, it is plausible that inhibition of these additional pathways may also contribute to the observed immunomodulatory and antitumor effects. Given the extensive crosstalk among STAT, NF-κB, metabolic, and stemness-associated pathways within the tumor microenvironment, the therapeutic efficacy of Niclosamide likely reflects coordinated modulation of multiple signaling networks rather than exclusive targeting of a single axis. Dissecting the relative contribution and potential synergy among these pathways will be an important direction for future investigation.

Our findings also provide mechanistic insight into the paradoxical role of IFN-γ in tumor immunity. While IFN-γ enhances T cell recruitment and activation, it simultaneously drives PD-L1 expression and T cell exhaustion, particularly under hypoxic conditions (**Figure 5**, **Figure 6**). This duality highlights the complexity of cytokine signaling within the TME and underscores the need for carefully balanced therapeutic strategies when modulating IFN-γ–dependent pathways.

Despite these insights, several limitations of this study should be acknowledged. First, our conclusions are primarily derived from *in vitro* and *ex vivo* systems, including tumor spheroids and T cell co-culture models. While these approaches recapitulate key features of hypoxia and immune suppression, *in vivo* validation will be required to fully assess therapeutic efficacy, immune dynamics, and safety in an intact immune system. In addition, Ki-67 staining could provide a complementary marker to further evaluate T cell proliferative capacity in our co-culture models.

Second, although robust immunomodulatory effects were observed in the MC38 model, additional validation across diverse tumor models and patient-derived clinical samples would strengthen the generalizability and translational relevance of our findings. Given tumor-type–specific differences in STAT signaling and hypoxic adaptation, such studies may help define the broader applicability of dual STAT1/3 targeting. Moreover, the potential synergistic effects of Niclosamide in combination with immunotherapeutic approaches - particularly immune checkpoint blockade - warrant systematic investigation *in vivo*.

Third, the RNA-seq analysis in this study was performed using a single biological sample per condition and was intended as an exploratory approach to identify candidate pathways regulated by IFN-γ and hypoxia. While quantitate real-time PCR was used to validate some of the genetic changes revealed by coherent pathway-level trends (**Figure 4**), future studies incorporating additional biological replicates will be necessary to strengthen statistical power and validate individual gene-level changes. In addition, although we observed a strong association between CXCL10 expression and T cell infiltration, this relationship remains correlative in the current study. We therefore explicitly acknowledge this limitation and propose the CXCL10–CXCR3 signaling axis as a potential mechanistic pathway for future investigation.

Finally, the precise molecular mechanism by which Niclosamide inhibits STAT1 and STAT3 remains to be fully elucidated. Based on prior studies, Niclosamide may interfere with upstream JAK kinase activity, STAT phosphorylation dynamics, or nuclear translocation, or indirectly modulate STAT signaling through metabolic or mitochondrial stress pathways, particularly under hypoxic conditions. Likewise, the molecular basis of reciprocal STAT1–STAT3 regulation within the tumor microenvironment—potentially involving competition for upstream kinases, transcriptional co-factors, or chromatin occupancy in response to IFN-γ and hypoxia—remains incompletely understood and merits further mechanistic investigation.

In conclusion, our study demonstrates that simultaneous targeting of STAT1- and STAT3-dependent IFN-γ signaling represents an effective strategy to overcome hypoxia-associated immune evasion. We identify Niclosamide as a dual STAT1/3 inhibitor that disrupts IFN-γ- and hypoxia-driven adaptive resistance, restores T cell function, and limits tumor plasticity. These findings provide a strong rationale for future *in vivo* studies and clinical evaluation of Niclosamide as a repurposed adjuvant agent in combination immunotherapy for refractory solid tumors.

## Data Availability

The RNA-seq datasets generated in this study have been deposited in the Gene Expression Omnibus (GEO) under accession number GSE320497. All other data supporting the findings of this study are available from the corresponding author upon reasonable request.
