# Supplementary material for: Identifying the therapeutic potential of niclosamide in overcoming IFN-gamma dependent cancer immune evasion in the tumor microenvironment
Source: Front Immunol. 2026 Mar 16;17:1761715. doi: 10.3389/fimmu.2026.1761715 (PMC13033776; doi:10.3389/fimmu.2026.1761715)
Supplement: Supplementary file 1 [file DataSheet1.pdf]

Supplemental Fig. 1 PD-L1 upregulation was observed on the surface of MC38 tumor cells with IFNγ treatment of different dosages and time periods.

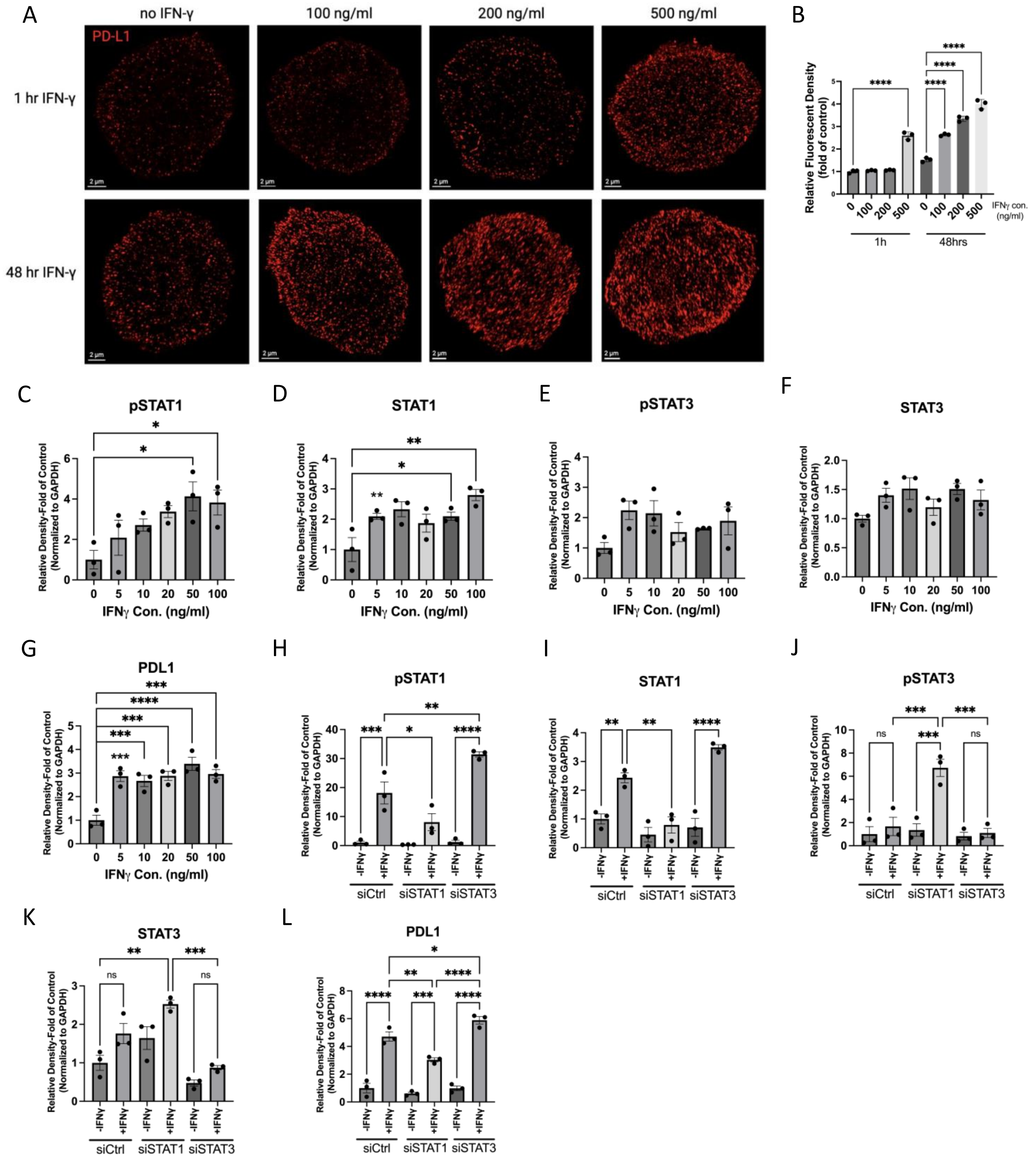

Supplemental Figure 1. PD-L1 upregulation was observed on the surface of MC38 tumor cells with IFNγ treatment of different dosages and time periods. (A)Confocal image of immune fluorescent staining of surface PDL1 on MC38 cells. (B) Quantification of fluorescent density to area in panel A. (C-G) Densitometric quantification of STAT1, STAT3, pSTAT1, and pSTAT3 normalized to GAPDH in Figure1K. (H-L) Densitometric quantification of STAT1, STAT3, pSTAT1, and pSTAT3 normalized to GAPDH in Figure1L. The results are expressed as the mean ± SEM of triplicate measurements in each group. *p<0.05, **p<0.01, ***p<0.001, ****p<0.0001.

Supplemental Fig. 2 PD-L1 upregulation by IFNγ was observed in induced MC38 tumor spheres, which exhibited more CSC features.

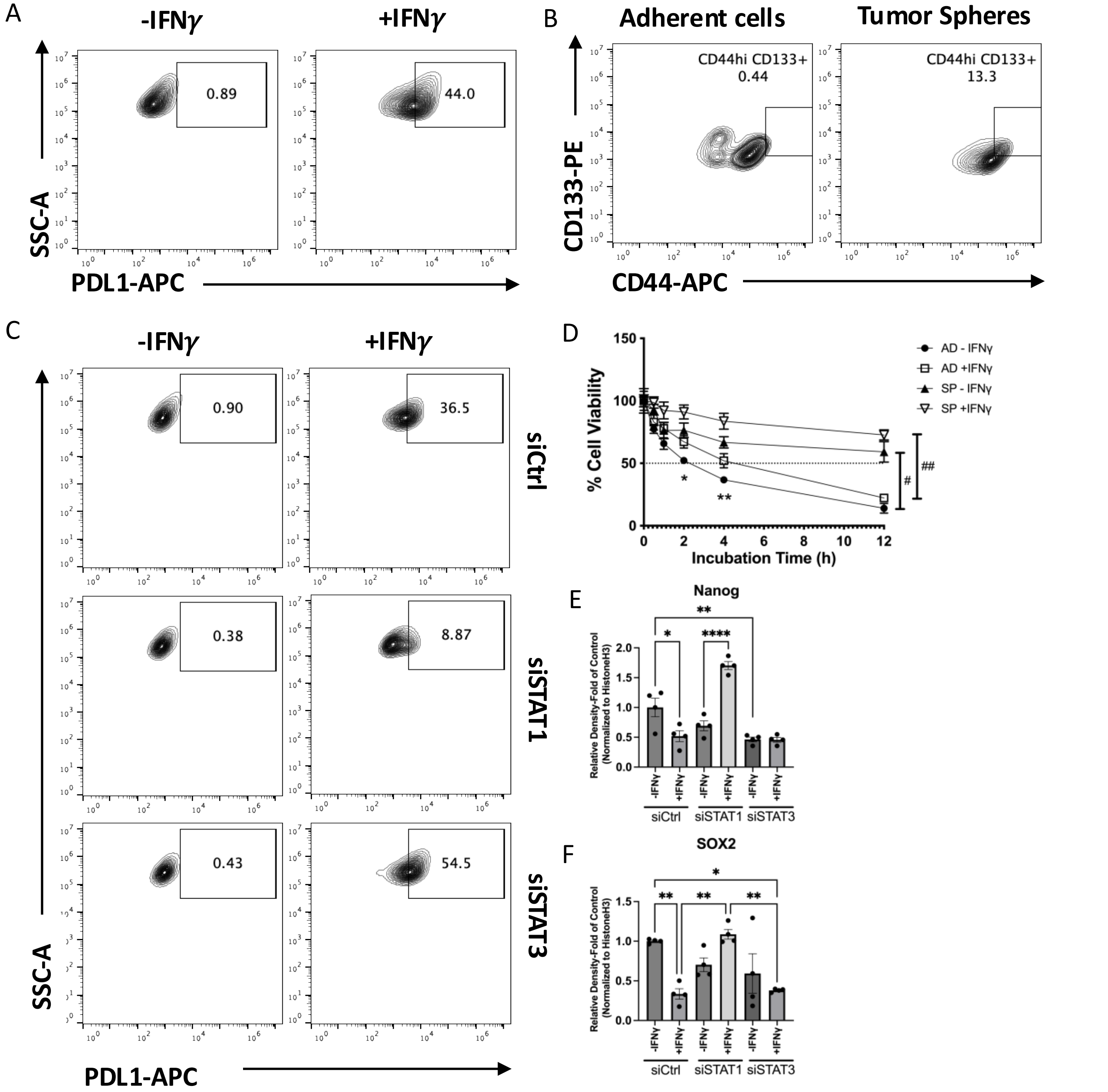

Supplemental Figure 2. PD-L1 upregulation by IFNγ was observed in induced MC38 tumor spheres, which exhibited more CSC features. (**A**) PD-L1 expression in MC38 tumor spheres level was measured by FACS. (**B**) Flow analysis revealed that the stem cell-like population was much more in tumor spheres compared to adherent cells. (**C**)The PDL1 expression in MC38 cells treated with siRNA of scramble control, STAT1 or STAT3. (**D**) Tumor spheres show more resistant to T cells compared to adherent MC38 cells when co-cultured with T cells. (**E-F**) Densitometric quantification of SOX2 and Nanog normalized to Histone H3 in Figure 2L. The results are expressed as the mean ± SEM of triplicate measurements in each group. *p<0.05, **p<0.01, ***p<0.001, ****p<0.0001.

Supplemental Fig. 3 Treatment of IFNγ affect tumor cells' viability while co-cultured with primary T cells.

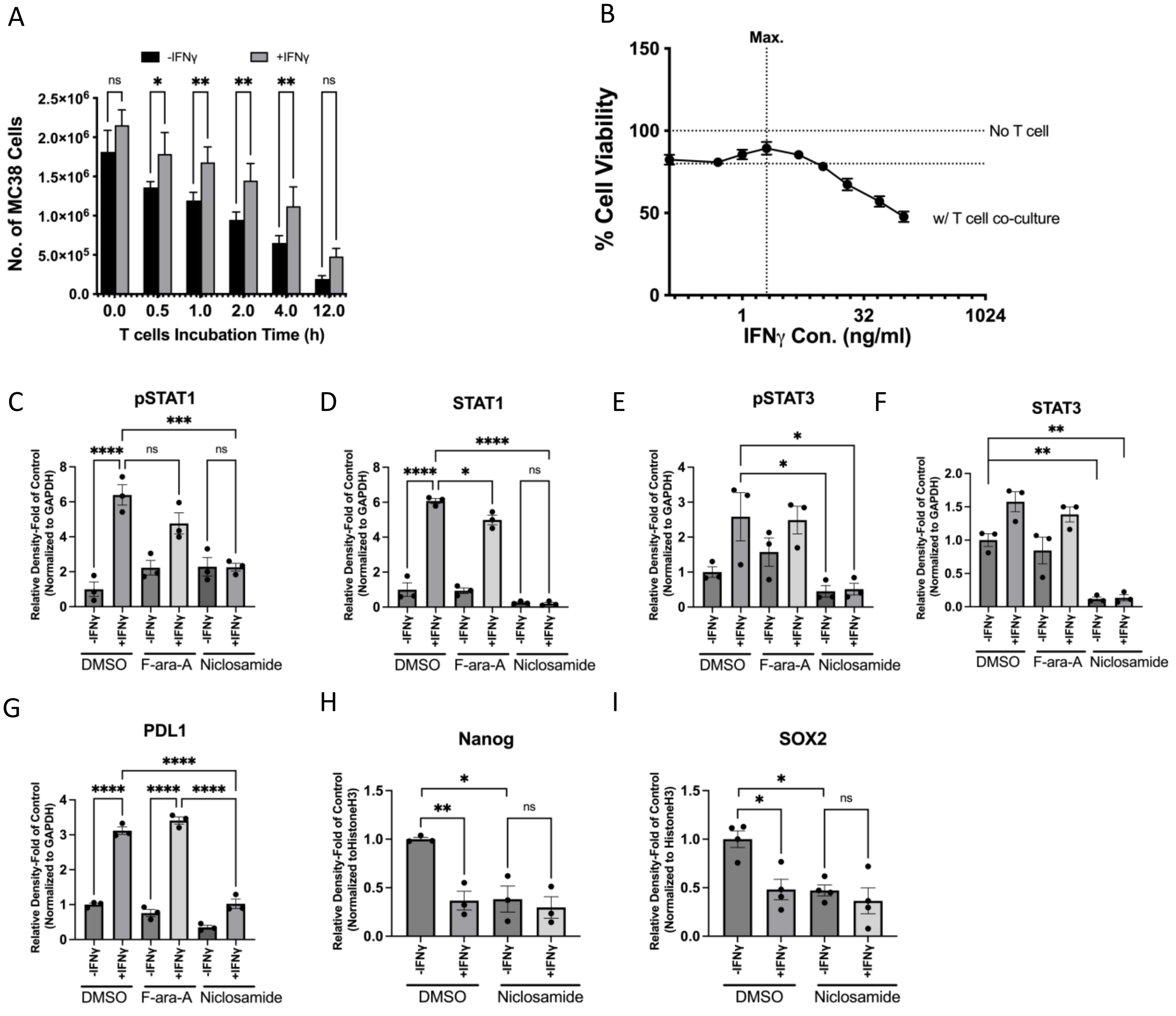

Supplemental Figure 3. Treatment of IFNγ affect tumor cells' viability while co-cultured with primary T cells.

(**A**) Live cells number of MC38 cells pre-treated with or without IFNγ and then co-cultured with primary T cells of different time period were measured by Trypan Blue assay. (**B)** Cell viability of MC38 cells pre-treated with different dose of IFNγ and co-cultured with primary T cells were measured by CCK8 assay. (**C-G**) Densitometric quantification of STAT1, STAT3, pSTAT1, and pSTAT3 normalized to GAPDH in Figure 3A. (**H-I**) Densitometric quantification of SOX2 and Nanog normalized to Histone H3 in Figure 3F. The results are expressed as the mean ± SEM of triplicate measurements in each group. *$p<0.05$, **$p<0.01$, ***$p<0.001$, ****$p<0.0001$.

Supplemental Fig. 4 Niclosamide reduce tumor spheres formation with or without IFNγ, while also decrease PDL1 expression in tumor spheres.

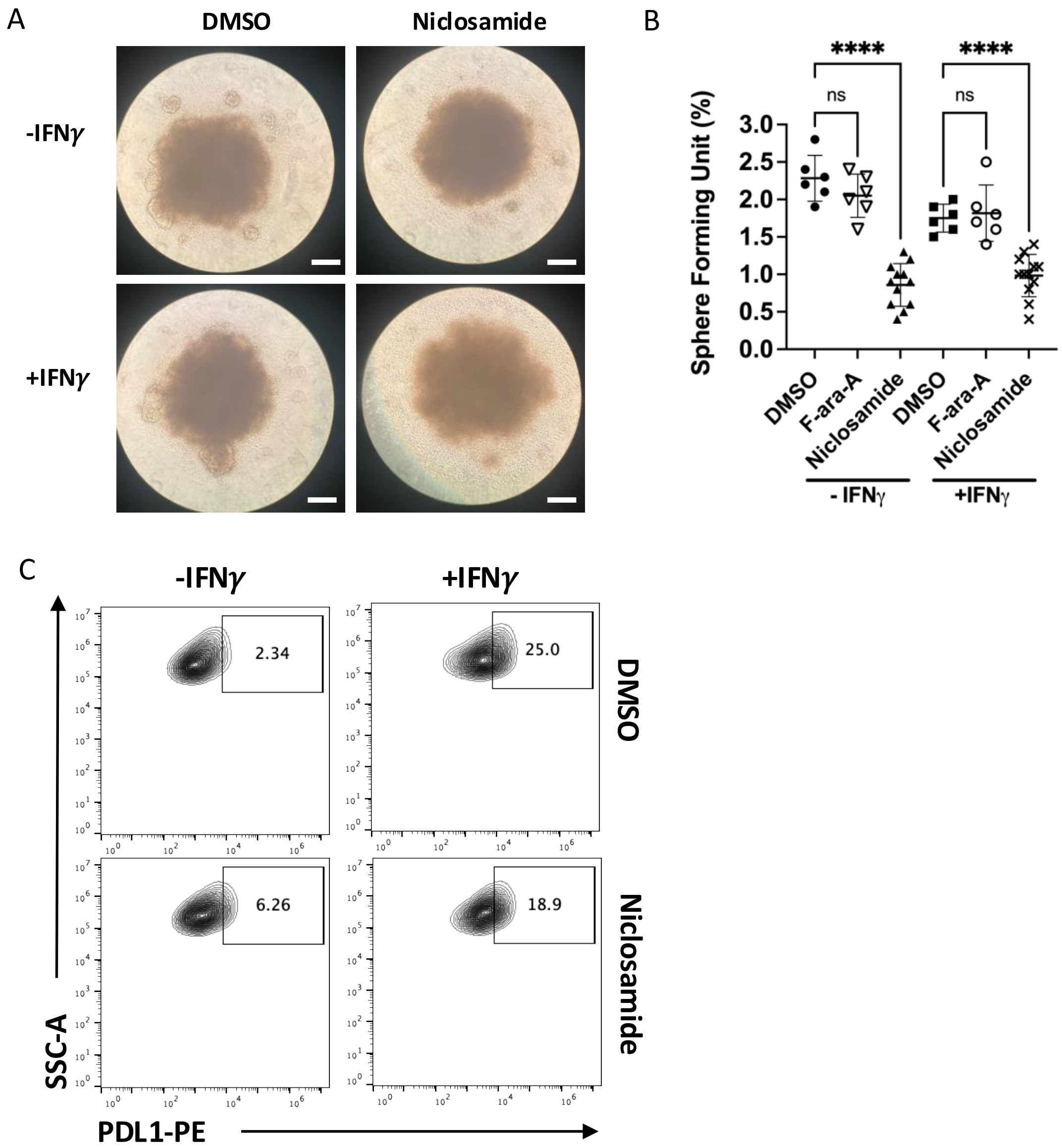

Supplemental Figure 4. Niclosamide reduce tumor spheres formation with or without IFNγ. (**A-B**) The tumor spheres forming unit in the MC38 cells were significantly reduced when treated with Niclosamide, but not with fludarabine (F-ara-A) scale bar: 200μm. (**C**) Niclosamide show partially blocking of the IFNγ induced up-regulation of PDL1 in MC38 tumor spheres. The results are expressed as the mean ± SEM of triplicate measurements in each group, ****$p<0.0001$.

Supplemental Fig. 5 Hypoxia condition interfere with IFNγ effect on tumor cells' expression of PD-L1 and STATs.

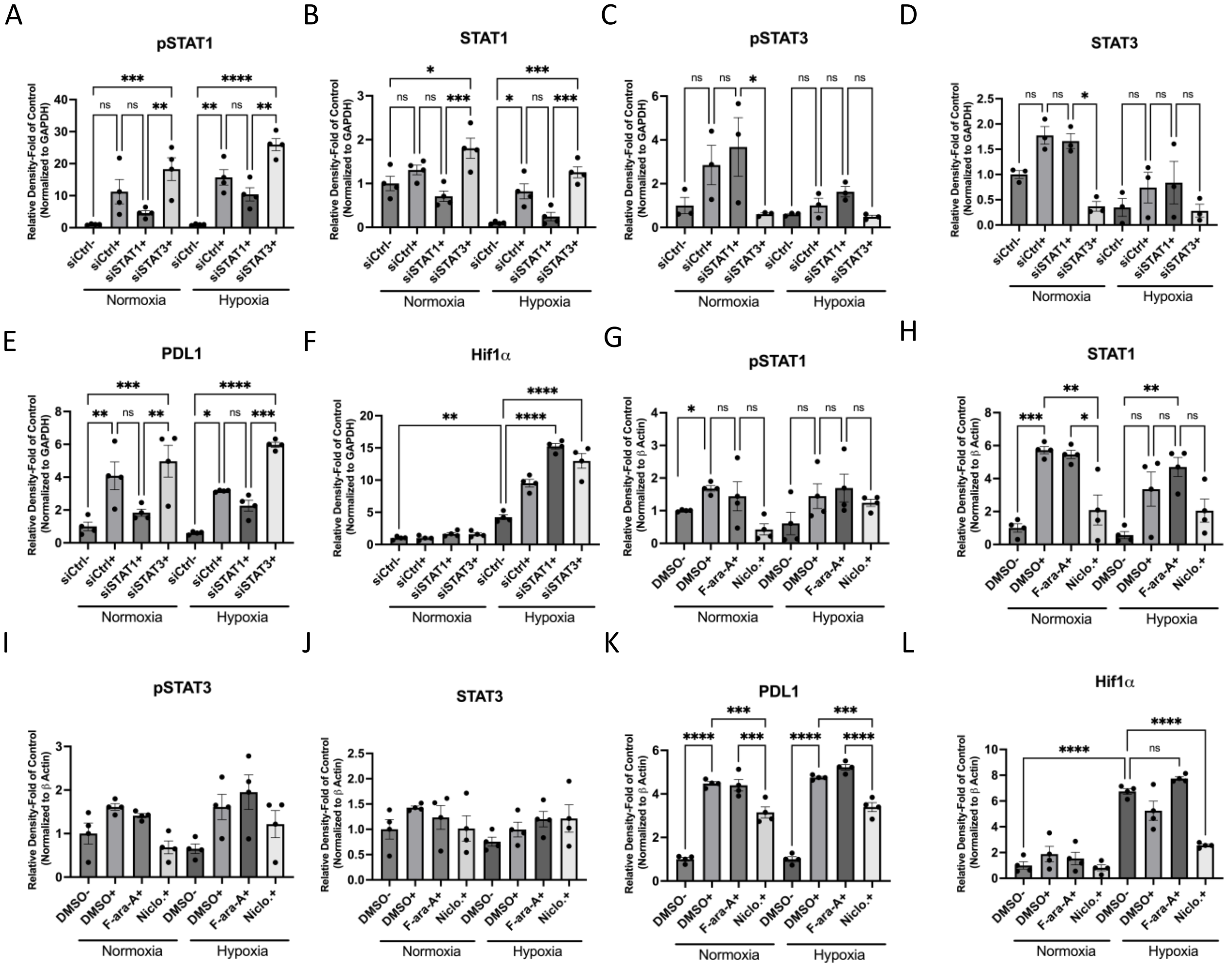

Supplemental Figure 5. Hypoxia condition interfere with IFNγ effect on tumor cells' expression of PDL1 and STATs.

(**A-F**) Densitometric quantification of STAT1, STAT3, pSTAT1, pSTAT3 and Hif1a normalized to GAPDH in Figure 5A. The siCtrl-, siCtrl+, siSTAT1+, siSTAT3+ on the X-axis indicating: MC38 cells treated with siCtrl and without IFNγ, treated with siCtrl and IFNγ, treated with siSTAT1 and IFNγ, treated with siSTAT3 and IFNγ. (**G-L**) Densitometric quantification of STAT1, STAT3, pSTAT1, pSTAT3 and Hif1a normalized to beta Actin in Figure 5B. The DMSO-, DMSO+, F-ara-A+, Niclosamide+ on the X-axis indicating: MC38 cells treated with 0.5% DMSO and without IFNγ, treated with 0.5%DMSO and IFNγ, treated with Fludarabine and IFNγ, treated with Niclosamide and IFNγ. The results are expressed as the mean ± SEM of triplicate measurements in each group. *p<0.05, **p<0.01, ***p<0.001, ****p<0.0001.

Supplemental Fig. 6 Mouse CD8+ T cells co-cultured with MC38 tumor cells were induced to massive exhaustion under hypoxia condition, and co-cultured with tumor spheroids reduced T cell proliferation.

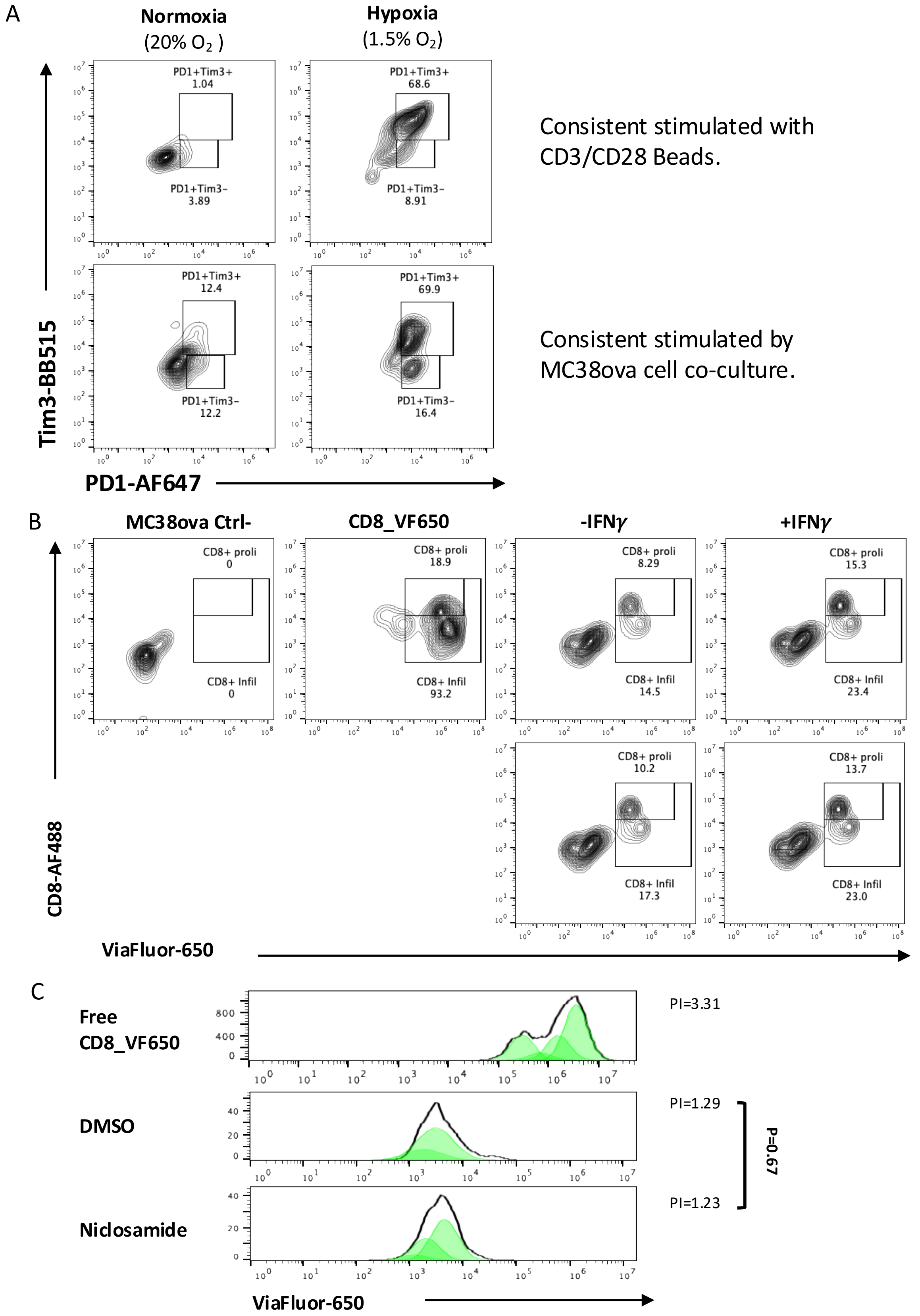

Supplemental Figure 6. Mouse primary CD8+ T cells co-cultured with MC38 tumor cells were induced to massive exhaustion under hypoxia condition, and co-cultured with tumor spheroids reduced T cell proliferation. (A) The flow analysis of two different protocol of inducing T cell exhaustion *in vitro*. Upper panel: the primary T cells were co-cultured with CD3/CD28 beads for 7 days. Lower panel: the primary T cells were co-coculture with MC38-OVA cells for 7 days.**(B)** Flow analysis of control MC38ova cells (without staining), CD8+T cells labeled with ViaFluor650, and CD8+ T cells get infiltrated into tumor spheroids, with or without IFNγ. The gating cell populations are CD8+ T cell get infiltrated into tumor spheres **(CD8+ Infil**), and CD8+ T cells get proliferated after infiltration **(CD8+ proli).** (C) Representative ViaFluor 650 dilution histograms showing that T cell proliferation Index was not changed by Niclosamide. Quantification of proliferation index (PI) across biological replicates is shown on the right; data are presented as mean ± SEM; p-values were calculated using permutation tests.

Supplemental Fig. 7 Mouse CD8+ T cells co-cultured with tumor spheroids with different STAT1/STAT3 Knock down shown different T cell infiltration.

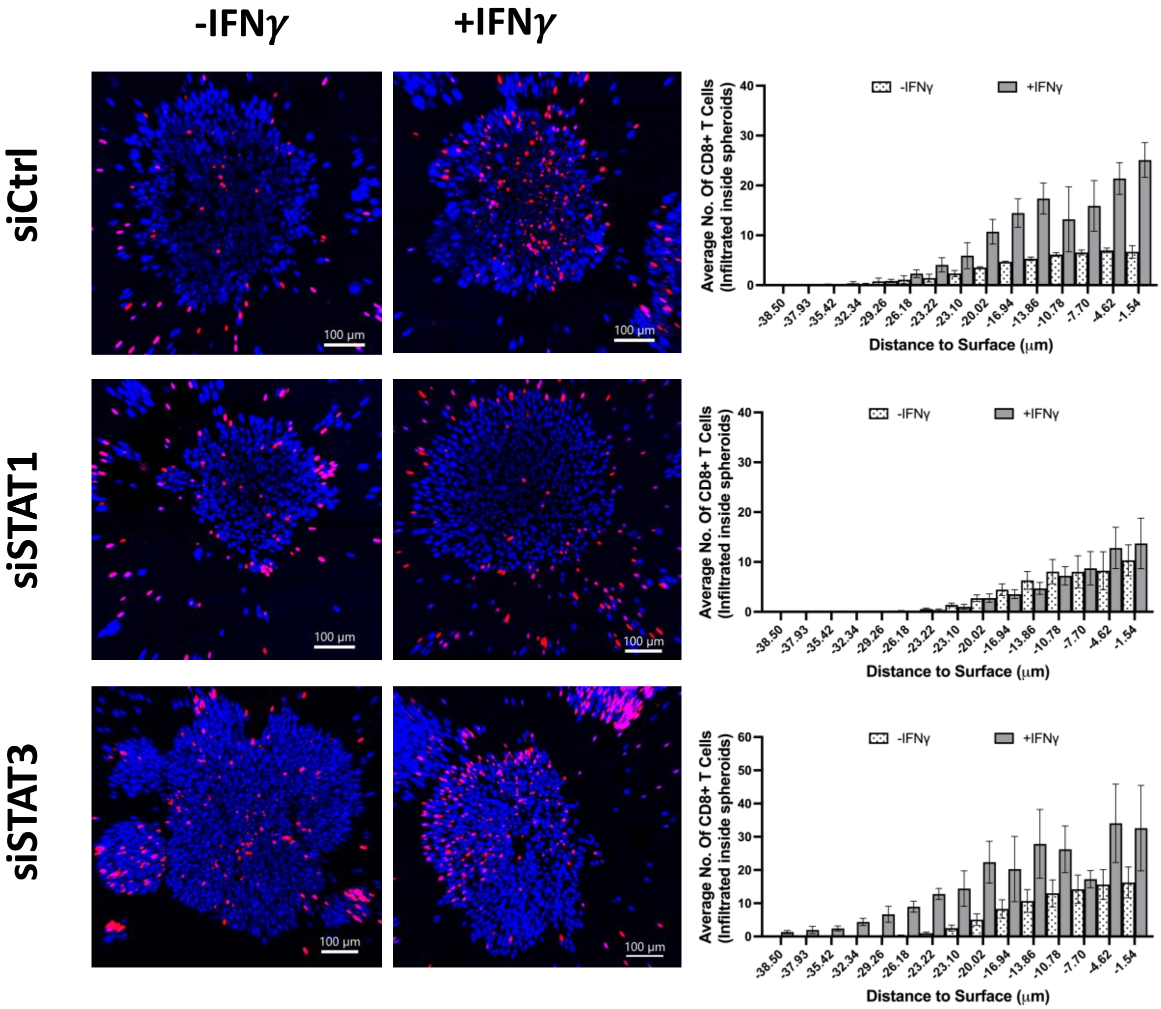

Supplemental Figure 7. Mouse primary CD8+ T cells co-cultured with tumor spheroids with different STAT1/STAT3 Knock down shown different T cell infiltration. Left panel: CD8+ T cell was tracked after labeling with the ViaFluor650. **Right panel:** T cell–to–tumor spheroid surface distances were quantified using the surface function in Imaris.
