# Supplementary material for: Identifying the therapeutic potential of niclosamide in overcoming IFN-gamma dependent cancer immune evasion in the tumor microenvironment
Source: Front Immunol. 2026 Mar 16;17:1761715. doi: 10.3389/fimmu.2026.1761715 (PMC13033776; doi:10.3389/fimmu.2026.1761715)
Supplement: Supplementary file 2 [file DataSheet2.pdf]

Supplemental Table 1

Name	Sequence
m GAPDH_F	CATCACTGCCACCCAGAAGACTG
m GAPDH_R	ATGCCAGTGAGCTTCCCGTTCAG
m PDL1_F	TGCGGACTACAAGCGAATCACG
m PDL1_R	CTCAGCTTCTGGATAACCCTCG
m STAT1_F	GCCTCTCATTGTCACCGAAGAAC
m STAT1_R	TGGCTGACGTTGGAGATCACCA
m STAT3_F	AGGAGTCTAACAACGGCAGCCT
m STAT3_R	GTGGTACACCTCAGTCTCGAAG
m SOX2_F	AACGGCAGCTACAGCATGATGC
m SOX2_R	CGAGCTGGTCATGGAGTTGTAC
m OCT4_F	CAGCAGATCACTCACATCGCCA
m OCT4_R	GCCTCATACTCTTCTCGTTGGG
m CD44_F	CGGAACCACAGCCTCCTTTCAA
m CD44_R	TGCCATCCGTTCTGAAACCACG
m CD133_F	CTGCGATAGCATCAGACCAAGC
m CD133_R	CTTTTGACGAGGCTCTCCAGATC
m Hif1a_R	CCTGCACTGAATCAAGAGGTTGC
m Hif1a_F	CCATCAGAAGGACTTGCTGGCT
m CXCL10_F	ATCATCCCTGCGAGCCTATCCT
m CXCL10_R	GACCTTTTTTGGCTAAACGCTTTC
m IL15_F	GTAGGTCTCCCTAAAACAGAGGC
m IL15_R	TCCAGGAGAAAGCAGTTCATTGC
m cMYC_F	TCGCTGCTGTCCTCCGAGTCC
m cMYC_R	GGTTTGCCTCTTCTCCACAGAC
m MCL-1_F	AGCTTCATCGAACCATTAGCAGAA
