# Supplementary material for: Identifying the therapeutic potential of niclosamide in overcoming IFN-gamma dependent cancer immune evasion in the tumor microenvironment
Source: Front Immunol. 2026 Mar 16;17:1761715. doi: 10.3389/fimmu.2026.1761715 (PMC13033776; doi:10.3389/fimmu.2026.1761715)
Supplement: Supplementary file 3 [file DataSheet3.pdf]

Supplemental Table 2

No	Antibody	Species	Application	Dilution	Manufacturer	Cat. No.
1	APC anti-mouse CD274 (B7-H1, PD-L1) Antibody	rat	flow, IF	1:100	biolegend	124312
2	FITC anti-mouse/human Ki-67 Antibody	rat	flow, IF	1:100	biolegend	151211
3	APC anti-mouse/human CD44 Recombinant Antibody	rat	flow	1:100	biolegend	163603
4	PE anti-mouse CD133 Antibody	rat	flow	1:100	biolegend	141203
5	BD Horizon™ BB515 Mouse Anti-Mouse CD366 (TIM-3)	rat	flow	1:100	BD Biosciences	567810
6	Alexa Fluor® 647 anti-mouse CD279 (PD-1) Antibody	rat	flow	1:100	biolegend	135230
7	Alexa Fluor® 488 anti-mouse CD8a Antibody	rat	flow	1:100	biolegend	100723
8	APC anti-mouse B7-H4 (B7S1, B7X) Antibody	Armenian Hamster	flow	1:100	biolegend	139407
9	PD-L1 (D4H1Z) Rabbit mAb #60475	Rabbit	WB	1:1000	Cell Signaling	60475T
10	β-Actin (8H10D10) Mouse mAb #3700	Mouse	WB	1:2000	Cell Signaling	3700S
11	GAPDH (D16H11) XP® Rabbit mAb #5174	Rabbit	WB	1:2000	Cell Signaling	5174S
12	Phospho-Stat3 (Tyr705) (D3A7) XP® Rabbit mAb #9145	Rabbit	WB	1:1000	Cell Signaling	9145S
13	Stat3 (79D7) Rabbit mAb #4904	Rabbit	WB	1:1000	Cell Signaling	4904S
14	Phospho-Stat1 (Tyr701) (58D6) Rabbit mAb #9167	Rabbit	WB	1:1000	Cell Signaling	9167S
15	Stat1 Antibody #9172	Rabbit	WB	1:1000	Cell Signaling	9172S
16	Sox2 (D6D9) XP® Rabbit mAb #3579	Rabbit	WB	1:1000	Cell Signaling	3579S
17	Nanog (D2A3) XP® Rabbit mAb #8822	Rabbit	WB	1:500	Cell Signaling	8822T
18	Histone H3 (1B1B2) Mouse mAb #14269	Mouse	WB	1:500	Cell Signaling	14269T
19	HIF-1α (D1S7W) XP® Rabbit mAb #36169	Rabbit	WB	1:500	Cell Signaling	36169T
20	Anti-rabbit IgG, HRP-linked Antibody #7074	Goat	WB	1:5000	Cell Signaling	7074S
21	Anti-mouse IgG, HRP-linked Antibody #7076	Horse	WB	1:5000	Cell Signaling	7076S
