# Supplementary figures and images for: Identifying the therapeutic potential of niclosamide in overcoming IFN-gamma dependent cancer immune evasion in the tumor microenvironment
Source: Front Immunol. 2026 Mar 16;17:1761715. doi: 10.3389/fimmu.2026.1761715 (PMC13033776; doi:10.3389/fimmu.2026.1761715)

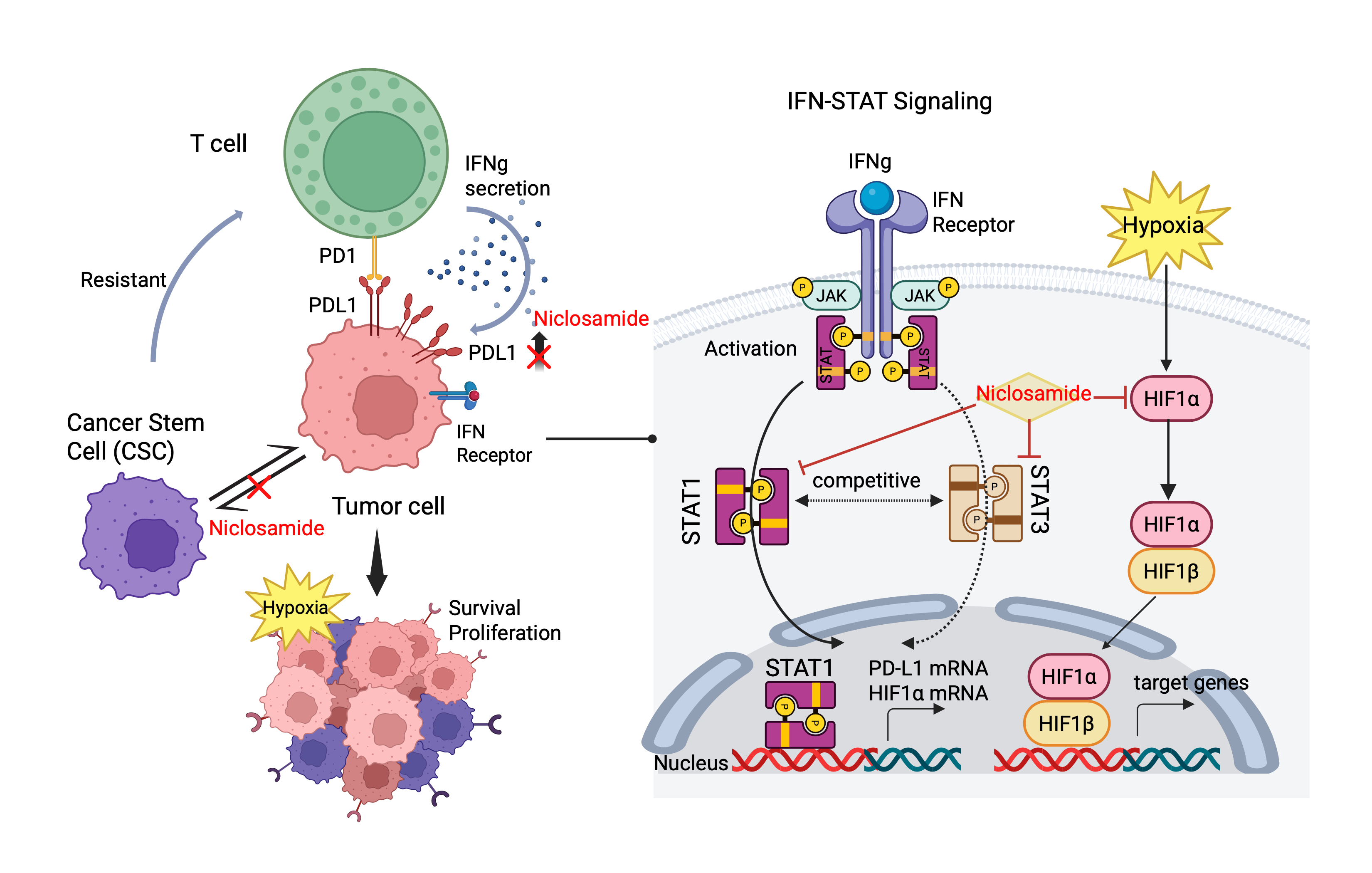

Supplement: Supplementary file 4 [file Image1.jpeg]
